# Virtual screening identifies broad-spectrum *β*-lactamase inhibitors with activity on clinically relevant serine- and metallo-carbapenemases

**DOI:** 10.1038/s41598-020-69431-y

**Published:** 2020-07-29

**Authors:** Francesca Spyrakis, Matteo Santucci, Lorenzo Maso, Simon Cross, Eleonora Gianquinto, Filomena Sannio, Federica Verdirosa, Filomena De Luca, Jean-Denis Docquier, Laura Cendron, Donatella Tondi, Alberto Venturelli, Gabriele Cruciani, Maria Paola Costi

**Affiliations:** 1 Department of Life Sciences, University of Modena and Reggio Emilia, Via Campi 103, 41125 Modena, Italy; 2 Department of Biology, University of Padua, Viale G. Colombo 3, 35121 Padua, Italy; 3 Molecular Discovery Limited, Centennial Avenue, Unit 501 Centennial Park, Borehamwood, Hertfordshire, WD6 3FG UK; 4 Department of Drug Science and Technology, University of Turin, Via Pietro Giuria 9, 10125 Turin, Italy; 5 Department of Medical Biotechnology, University of Siena, Viale Bracci 16, 53100 Siena, Italy; 6 TYDOCK PHARMA S.R.L., Strada Gherbella 294/b, 41126 Modena, Italy; 7 Department of Chemistry, Biology and Biotechnology, University of Perugia, Via Elce di Sotto 8, 06123 Perugia, Italy; 8 Present Address: Department of Drug Science and Technology, University of Turin, Via Pietro Giuria 9, 10125 Turin, Italy

**Keywords:** Drug discovery, Virtual screening

## Abstract

Bacteria are known to evade β*-*lactam antibiotic action by producing β-lactamases (BLs), including carbapenemases, which are able to hydrolyze nearly all available β-lactams. The production of BLs represents one of the best known and most targeted mechanisms of resistance in bacteria. We have performed the parallel screening of commercially available compounds against a panel of clinically relevant BLs: class A CTX-M-15 and KPC-2, subclass B1 NDM-1 and VIM-2 MBLs, and the class C *P. aeruginosa* AmpC. The results show that all BLs prefer scaffolds having electron pair donors: KPC-2 is preferentially inhibited by sulfonamide and tetrazole-based derivatives, NDM-1 by compounds bearing a thiol, a thiosemicarbazide or thiosemicarbazone moiety, while VIM-2 by triazole-containing molecules. Few broad-spectrum BLs inhibitors were identified; among these, compound **40** potentiates imipenem activity against an NDM-1-producing *E. coli* clinical strain. The binary complexes of the two most promising compounds binding NDM-1 and VIM-2 were obtained at high resolution, providing strong insights to improve molecular docking simulations, especially regarding the interaction of MBLs with inhibitors.

## Introduction

β-lactam antibiotics are the most commonly used antimicrobials in the treatment of bacterial infections because of their spectrum of activity, clinical effectiveness and safety profile^1^. However, the continuous dissemination of BLs with an extended spectrum of activity, that is, Extended-Spectrum BLs (ESBLs) and carbapenemases, has deeply compromised their efficacy over the years and represents a real menace to public health^2^.

Notably, resistance to β-lactam antibiotics in Gram-negative bacteria mostly relies on the production of BLs, many of which are plasmid-mediated^3^. Thus, acquired BLs, due to their association with mobile genetic elements commonly encoding other resistance determinants, have led to the emergence of multi-drug resistant (MDR) and extensively-drug resistant (XDR) strains^4^. Moreover, the evolutionary potential of BLs and their presence in the environmental resistome is responsible for the mobilization and acquisition of new genes in response to the introduction of new β-lactams, such as carbapenems, the last resort antibiotics towards which resistance rates are dramatically increasing^4–6^.

BLs are categorised in two distinct families, according to their catalytic mechanism, and further divided into structural classes (Ambler’s classification^7^): class A, C, and D serine β-lactamases (SBLs), which utilize a catalytic serine for β-lactam hydrolysis, and class B metallo-β-lactamases (MBLs), which, in turn, require divalent zinc ions for catalysis^8,9^.

Class A includes both plasmid-mediated and chromosomally encoded β-lactamases, such as ESBLs, e.g. CTX-M-15, and carbapenemases, e.g. KPC-2^5,10^.

Class C enzymes, mostly chromosomally encoded (e.g. AmpC), are resistant to many suicide BLs inhibitors (BLIs), such as clavulanic acid^11^, but more efficiently inhibited by recently-approved inhibitors, such as avibactam or taniborbactam^12^. Some class D members (OXA-24 and -48) can hydrolyse last generation cephalosporins and carbapenems^13,14^.

Finally, subclass B1 MBLs inactivate nearly all β-lactam antibiotics, with the sole exception of monobactams. This class includes variants such as IMP-1, VIM-2 and NDM-1, whose worldwide emergence has characterized recent decades. While BLI-containing combinations are commercially available for the treatment of infections caused by SBL-producing organisms, none are currently available for MBL-producing isolates, which represent a serious threat to human health, due to their commonly broad antibacterial resistance profile^15,16^. Such infections, indeed, when caused by MBL-producing MDR or XDR strains, often respond only to tigecycline or polymyxins, the latter suffering from important tolerability issues^17^. The current situation highlights the importance of the continuous development of novel BLIs, preferably with broad-spectrum activity^18–20^. Unfortunately, the design of a BLI blocking SBLs and MBLs is extremely challenging, mainly for the significant differences between BLs, in terms of structure and mechanism of hydrolysis (Fig. S1). Currently, only one agent, i.e. taniborbactam, able to inhibit both SBLs and MBLs, has reached the stage of clinical development in combination with cefepime^21^.

With the aim of identifying novel chemotypes able to inhibit both SBLs and MBLs, we have implemented an “in parallel” in silico*/*in vitro approach to screen a large database of commercially available compounds against a panel of clinically relevant enzymes. Computational biology and molecular modelling methods have proven, over the years, to be powerful tools in drug design in general^22,23^ and in the identification of scaffolds for the development of new potential BLIs^24–27^. Nevertheless, we must acknowledge that the design of non-covalent inhibitors for SBLs, which are known to be targeted by covalent inhibitors mimicking the tetrahedral transition state, remains quite challenging^25,28,29^. Here, we present the identification of a series of non-covalent scaffolds targeting SBLs and concomitantly MBLs, through a large virtual screening study, in vitro validation, microbiological analysis and X-ray crystallography.

## Results and discussion

A commercially available library, previously filtered for drug-likeness properties, was screened in silico for potential ligands of CTX-M-15 ESBL and KPC-2 carbapenemase, and of NDM-1 and VIM-2 MBLs. The results from each screening, i.e. the most interesting molecules for each BL according to the pose in the binding site, the number of hydrogen bonds formed with the surrounding residues and the chemical diversity, were carefully analysed and guided the final candidate selection. A total of 66 molecules were purchased and subsequently tested in enzyme assays to evaluate their potential to inhibit the four above-mentioned enzymes, with the addition of AmpC from *Pseudomonas aeruginosa*, as a representative of class C BLs (structure and inhibition profile of the selected compounds are reported in Table 1 and Table S1).

**Table 1:**
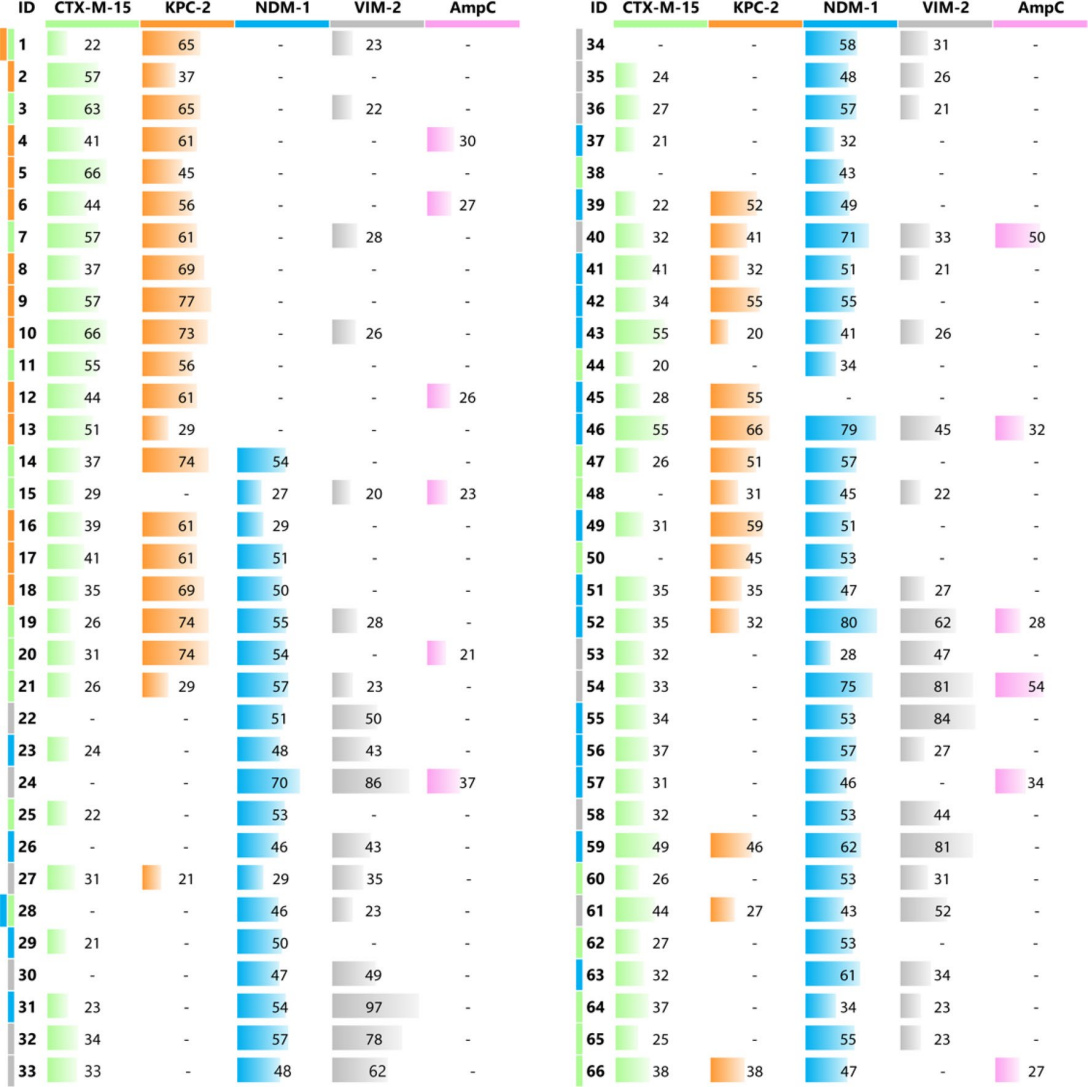
Library inhibition profile.

**Chart 1:**
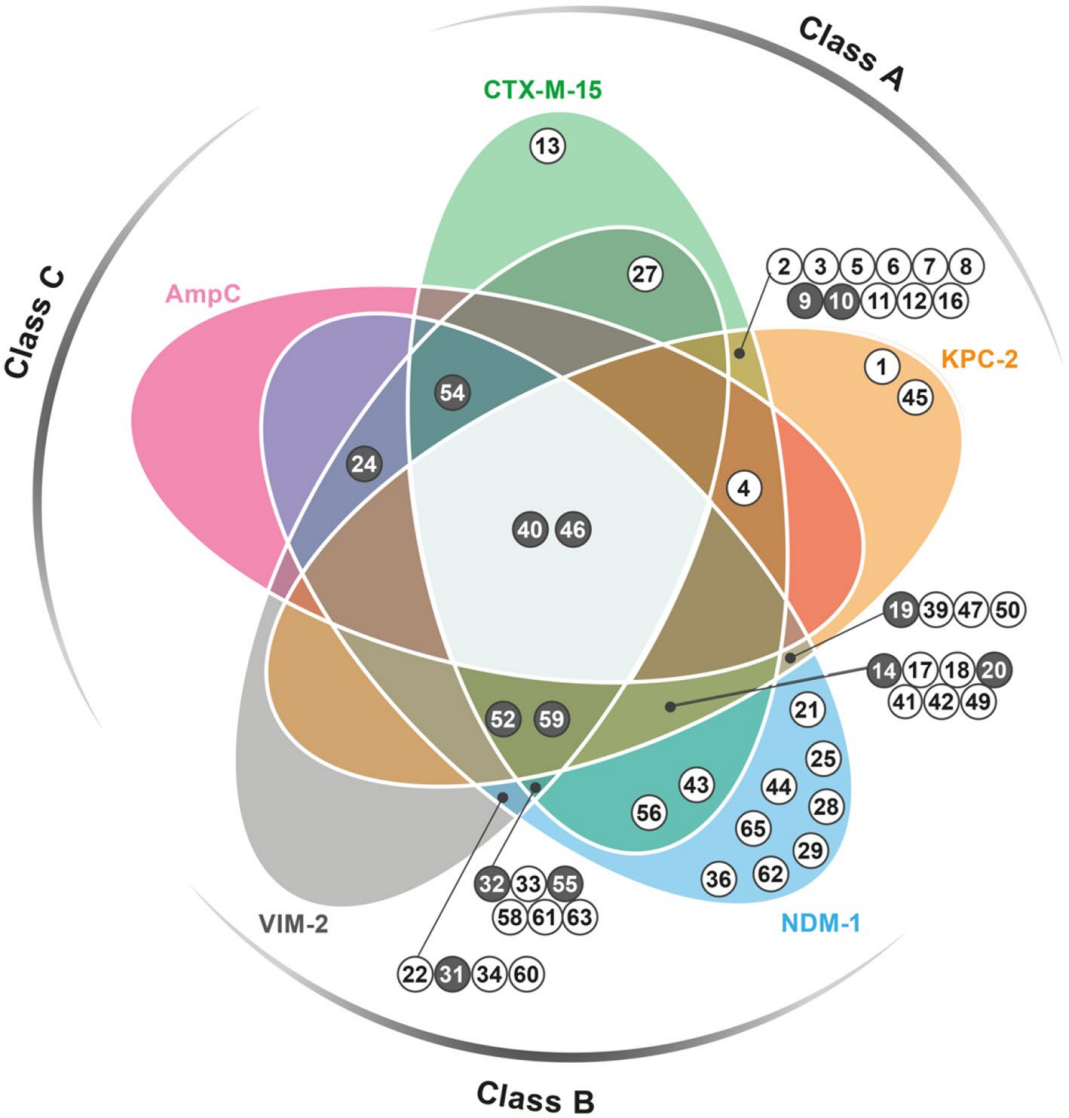
Venn diagram reporting the inhibition profile of active compounds towards the BLs panel. Each coloured area refers to a specific BL: CTX-M-15 green, KPC-2 orange, NDM-1 blue, VIM-2 grey, AmpC, pink. Compounds showing inhibition ranging 50–70% or > 70% towards, at least, one target are represented by white-filled and grey-filled circles, respectively, and are located under the curve belonging to that specific enzyme. Those exerting a minimum 30% inhibition towards other BLs are located in overlapping areas. The inhibition percentage exact values are reported in Table 1.

**Figure 1:**
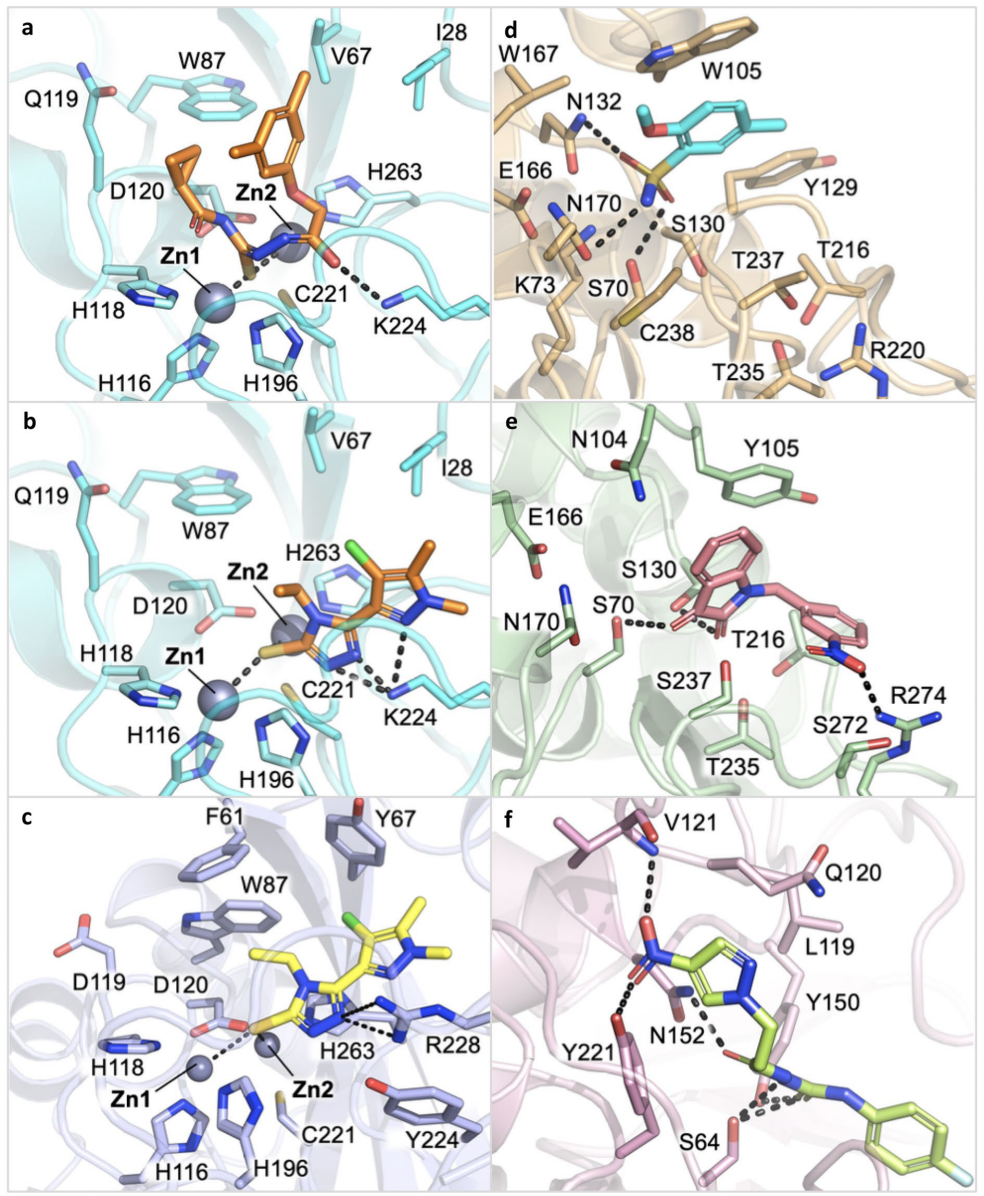
Docking pose of some of the most promising compounds in the corresponding BL binding site. (**a**) Compound **52** in NDM-1 (80% inhibition; protein PDB code 5zge). (**b**, **c**) Compound **24** in NDM-1 (70%; 5zge) and VIM-2 (86%; 2yz3), respectively. (**d**) Compound **9** in KPC-2 (77%; 3dw0). (**e**) Compound **7** in CTX-M-15 (57%; 4hbt). (**f**) Compound **54** in AmpC (51%; 1kdw). Pictures were prepared with Pymol v1.7.6.4.

The inhibitory activity of the compounds was evaluated towards a panel of relevant BLs (the percentage of enzyme inhibited fraction is reported except when ≤ 20% and in this case is indicated by a hyphen). The same enzyme colour code is used in both Fig. 1 and Chart 1. The colour next to the ID code indicates for which BL the compound was originally selected in silico.

To better appreciate the inhibition profile of the entire library, we classified the compounds in two categories having, respectively, inhibition ranging 50–70% or > 70% towards at least one BL (Chart 1). The diagram provides a rapid glimpse of the obtained results and of the cross-activity of the compounds towards the different BLs.

The structure of compounds showing at least 70% inhibition, when tested at 200 μM, are shown in Table 2. The docking poses of some of them for each targeted BL are shown in Fig. 1.

**Table 2:**
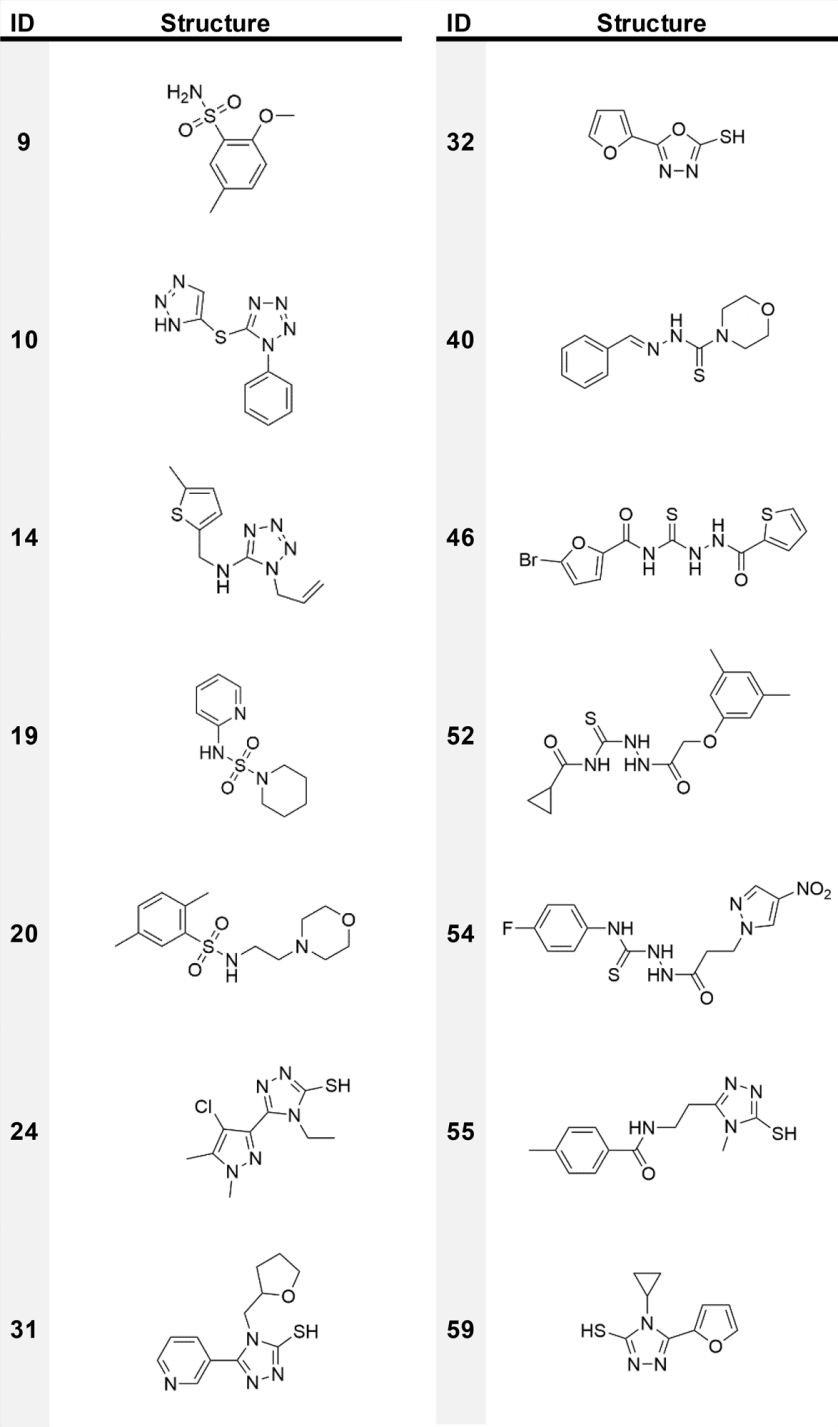
Chemical structure of the compounds showing at least 70% inhibition towards one BL.

### NDM-1 inhibition

Twelve molecules were selected in silico as possible MBL NDM-1 inhibitors. Eleven of these demonstrated good inhibition potency (≥ 50%), giving a VS success rate of 55% (*For VS success rate we intend, in this paper, the percentage of compounds having an inhibition percentage* ≥ *50% towards a specific BL, with respect to those selected as possibly active for that BL)*. Another twenty molecules, which were selected to inhibit SBLs, also performed well (≥ 50%) against NDM-1. Of these thirty-one compounds, **24, 40, 46, 52** and **54,** showed an inhibition activity higher than 70% and were all originally identified as potential ligands of a MBL. All the last present a thiol, a thiosemicarbazide or thiosemicarbazone moiety able to undergo an iminothiol/thiourea tautomerization and to generate a thiol group^30,31^. It must be noted that the p*K*_a_ of sulfhydryl groups in MBL decreases by about two orders of magnitude, due to the zinc presence, leading to deprotonation and strong coordination of the metal ions^32,33^. In the active compounds, thiols and thioureas are often associated to other electron pair donors that can complement the electron request of the MBL binding site^15,25^.

Looking at compound **52** in NDM-1 (Fig. 1a), this active site’s requirement appears properly fulfilled, as also confirmed by the good superposition of the central core of the molecule with the H-bond acceptor Molecular Interaction Field (MIF; Fig. S2, red contour).

Other compounds found active towards NDM-1 present diverse moieties having electron pairs such as carboxylate, sulfhydryl-triazole, benzo-triazole, nitro-triazole, amido-tiazole, sulfhydryl-tiadiazole, sulfone, previously reported among NDM-1 inhibitors^15,34–36^.

### VIM-2 inhibition

The VS success rate against VIM-2 was 43%. Overall, ten molecules were confirmed to be active against VIM-2 with an inhibitory activity ≥ 50% and, of these, six showed an inhibition higher than 70%. Four molecules were originally selected for their potential binding to NDM-1, which belongs to the same subclass.

The most potent compound is **31**, which demonstrates an inhibition percentage of 97%. Its predicted orientation in the VIM-2 active site is shown in Fig. S3. Interestingly, the sulfhydryl-triazole moiety of compound **31** is present in several of the most active VIM-2 inhibitors, **24, 55** and **59**. This class of molecules has been previously reported to inhibit several clinically relevant MBLs^35^.

Compounds **24** and **54** showed an inhibition ≥ 70% towards both VIM-2 and NDM-1 (Fig. 1b, c, S4, S5). While coordinating the zinc ions, compounds **24** and **54** in NDM-1 interact, respectively, with Lys224/211 and Asn233/220, crucial residues involved in β-lactam recognition and hydrolysis^37^. Notably, the conserved Asn233/220 forms an oxyanion hole together with Zn1 and is involved in the polarisation of the lactam carbonyl upon binding, thus facilitating the nucleophilic attack by the activated hydroxide. Therefore, targeting these residues might be critical for the design of potent NDM-1 inhibitors^15^. Interestingly, the mentioned residues appear involved in H-bond networks with many other compounds here identified as active towards MBLs.

Other compounds that can inhibit VIM-2 within a 50–70% range include **22**, **33**, **52** and **61**.

### KPC-2 inhibition

A good VS success rate was obtained for KPC-2 with ten compounds validated in vitro as active, out of the thirteen selected (77% success rate). On the whole, including those molecules selected for the other class A SBL, CTX-M-15, twenty-two molecules showed inhibition between 50 and 70% and five > 70%. The best performing compound, **9**, is shown in Fig. 1d and S6. Compounds bearing triazole, tetrazole and sulphonamide moieties were mainly found. With respect to the scaffolds that are preferred by NDM-1 and VIM-2, KPC-2 inhibitors miss the sulfhydryl group. Nevertheless, they present electron pair donors that can interact with residues Ser70, Lys73, Ser130 and Thr237 in the active site. This common feature may explain the cross reactivity of some compounds selected for SBLs, but able to target MBLs as well^38^.

### CTX-M-15 inhibition

The screening performance obtained for CTX-M-15, with only three molecules active in vitro*,* was relatively low, with a 14% success rate. The active compounds, **3**, **7** and **11**, have quite different structures and a poor inhibition percentage (63% and lower). The best pose, according to the score, the number of H-bonds and the superimposition with the Molecular Interaction Fields, is assumed by compound **7** (Figs. 1e and S7), which forms H-bonds with Ser70 and Ser130 through the indole dione moiety, and with Arg274 through the nitro group. Hydrophobic contacts are formed between the indole and Tyr105. Other molecules among those that were predicted to be active for KPC-2 and NDM-1 also showed some inhibition *vs* CTX-M-15. Overall, thirteen of these molecules, which present a wide range of different chemical structures, were detected.

### AmpC inhibition

As mentioned, all compounds were also tested *vs* AmpC, a class C SBL against which only last generation inhibitors, such as the recently approved avibactam, are effective. Compounds **40** and **54**, which were originally selected for VIM-2, were able to exert 50% inhibition on AmpC. The pose of compound **54** is shown in Fig. 1f and S8. The complex is stabilised by a number of H-bonds that are formed by the nitro moiety with Val121 and Tyr221, by the carbonyl with Asn152 and by the thiosemicarbazide with the catalytic Ser64. As mentioned above, p*K*_a_ predictions, which were performed using Moka^39^, and the absence of zinc ions, indicate that the most favoured tautomer of the thiosemicarbazide should be the thioureidic form.

Compound 7 chemical structure, not reported in Table 2, is shown in Table S1.

### Spectrum of inhibition of the identified MBL inhibitors

All compounds found to be active towards VIM-2 are active against NDM-1 as well (Chart 1); in particular compounds **24** and **54** showed the highest inhibition percentage towards both proteins. Interestingly, eight compounds were active only against NDM-1. With respect to VIM-2, NDM-1 has a slightly more extensive active site^40^, which derives primarily from the orientation of the two loops L3 (residues 61–67/65–74) and L10 (residues 221–240/205–228), involved in substrate recognition and hydrolysis in subclass B1 MBLs (Fig. S9). The most important residues on loop L10 are Arg228/185 and Asn233/190 in VIM-2^41^, Lys224/211 and the same Asn233/210 in NDM-1. Interestingly, Lys224/211 is conserved in all subclass B1 representatives, apart from the VIM variants. The capability of many of the identified candidates to target these residues might enlarge the compound inhibition profile towards different MBLs.

### Spectrum of inhibition of the identified SBL inhibitors

A number of compounds has been found active towards both class A BLs, CTX-M-15 and KPC-2 (Chart 1). The superposition of the proteins highlights the similarity of the active sites: only five residues differ, displaying, indeed, quite conservative substitutions (Fig. S10).

### X-ray crystallography of compounds 24 and 31 binary complexes

**Figure 2:**
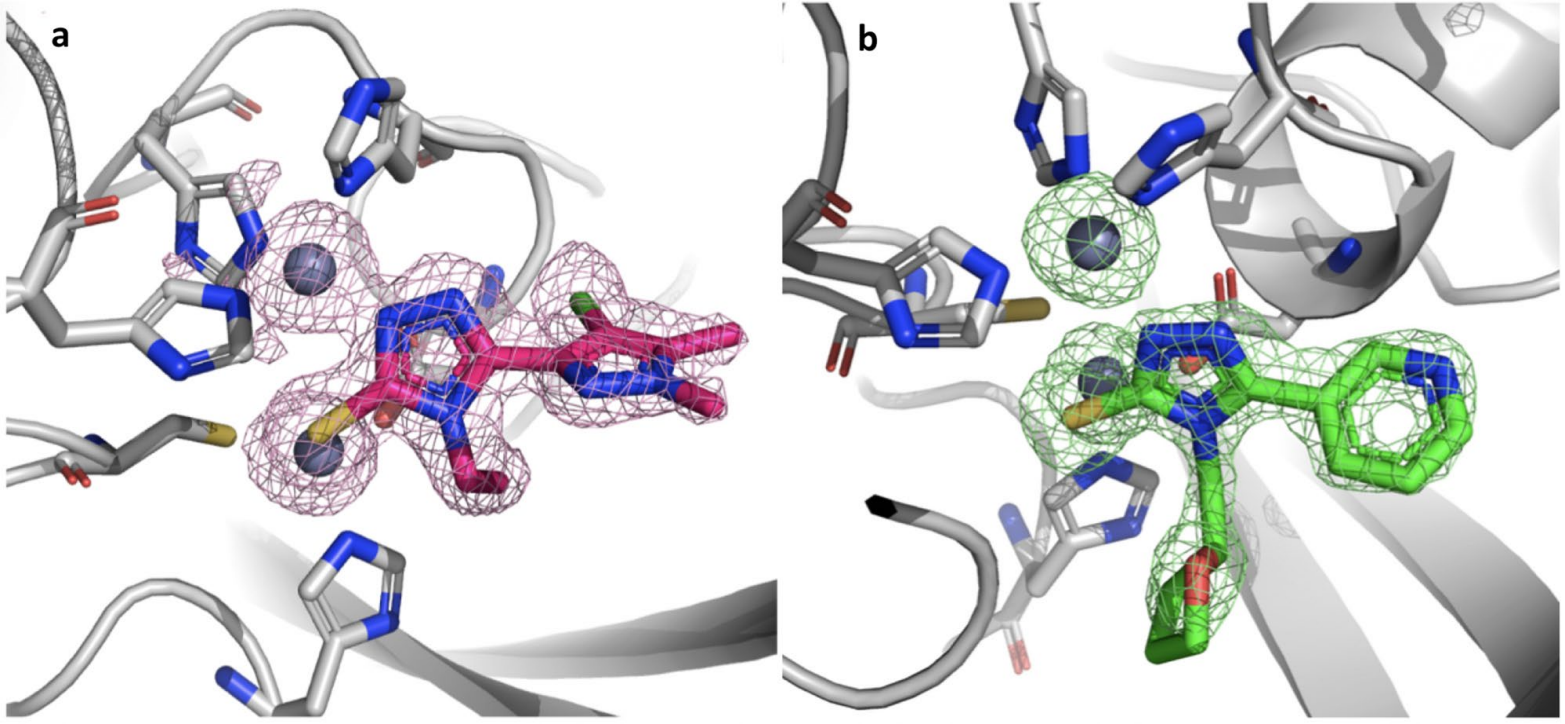
Binary complexes of compound **24** binding to VIM-2 and of compound **31** binding to NDM-1. (**a**) Omit map of **24**:VIM-2 binary complex shown at 3.5 σ contour level. (**b**) **31**: NDM-1 binary complex. The ligand and the residues lining the pocket are shown as capped sticks, the Zn ions as grey spheres.

**Figure 3:**
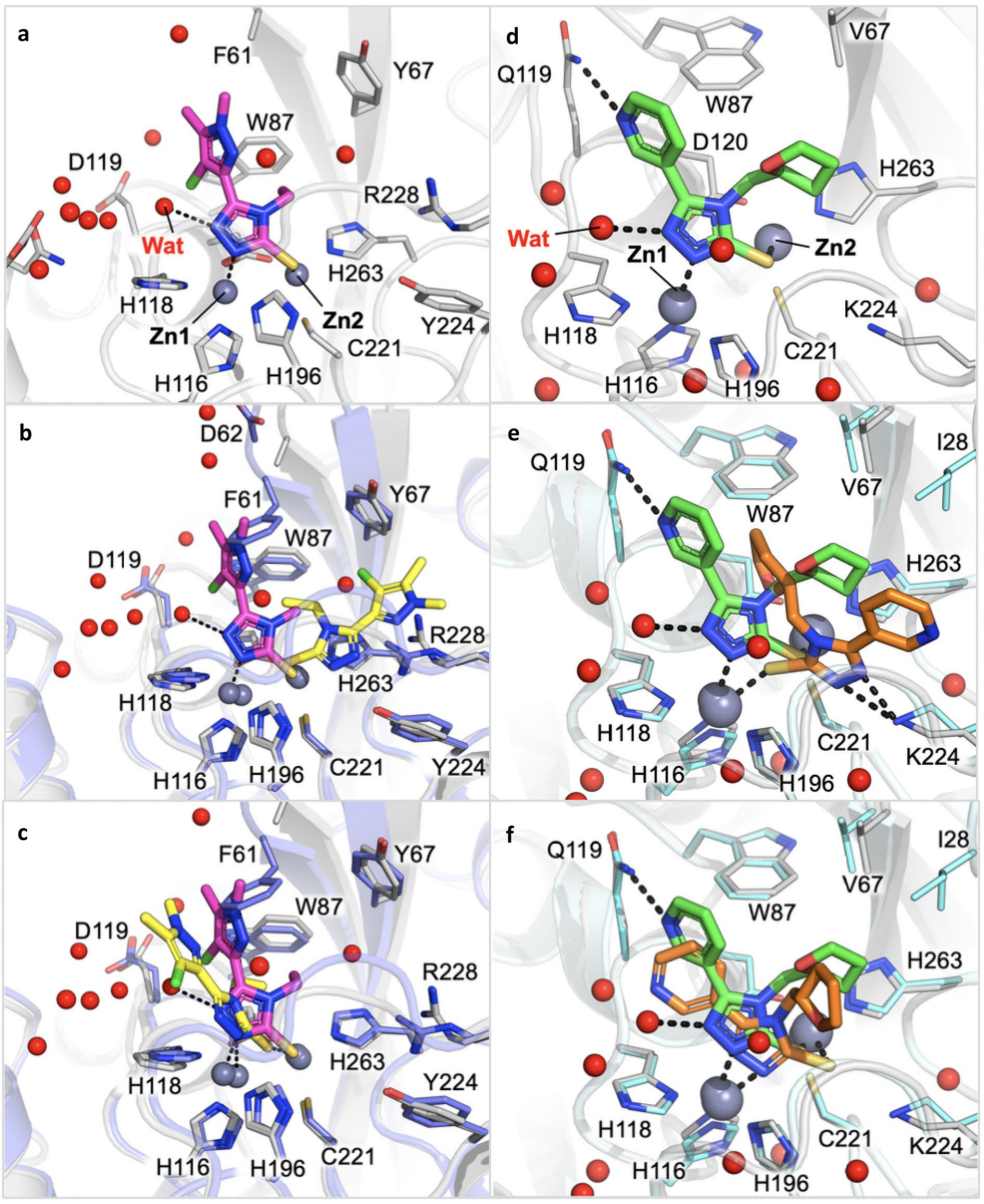
(**a**) Binary complex of compound **24** binding to VIM-2. (**b**) Superposition of the crystallographic (magenta) and of the originally selected docking pose (yellow) of compound **24** in VIM-2 binding site. (**c**) Superposition of the crystallographic (magenta) and of the most similar docking pose (yellow) of compound **24** in VIM-2 binding site. The protein of the binary X-ray complex is coloured grey, while the structure used for docking simulation is violet (**d**). Binary complexes of compound **31** binding to NDM-1. (**e**). Superposition of the crystallographic (green) and of the best ranked docking pose (orange) of compound **31** in NDM-1 binding site. The protein of the binary X-ray complex is coloured grey, while the structure used for docking simulation is cyan (**f**). Superposition of the crystallographic (green) and of the originally selected, and most similar docking pose (orange), of compound **31** in NDM-1 binding site. Red spheres correspond to water molecules.

High-resolution structures of compounds **24** and **31** in complex with VIM-2 and NDM-1 MBL have been determined by single-crystal X-ray diffraction at 1.60 and 1.38 Å resolution, respectively (Fig. 2, Table S4, S5, Fig. 3a, d). Two molecules per asymmetric units are present in both experimental models. Indeed, the two protein chains are present as unique elements in the crystal a.s.u., and the main features of the active site, as well as compound binding orientation, are conserved among the complexes.

With respect to the apo form of the enzyme the residues defining the active site undergo very minor conformational changes, as well as the two loops, L3 and L10, which define the entrance of the active site. In particular, in the case of NDM-1 they experience a slight opening upon binding of the inhibitors, while in VIM-2 their position appears quite conserved. The two zinc ions move away from each other a little, to better coordinate the ligand electron pair donors. A slight movement in the order of 0.5–0.8 Å is observed with respect to the apo structure. Inspection of the difference density and OMIT maps undoubtedly identifies the ligand presence and orientation in the modelled state.

In VIM-2 binding site compound **24** orients the central sulfhydryl-triazole-based scaffold deep in the pocket, with one of the triazole nitrogen contacting Zn1, while Zn2 is coordinated by the sulfhydryl group. The second triazole nitrogen interacts with a crystallographic water molecule. Surprisingly, no other direct contact is formed between the inhibitor and the surrounding residues. (Fig. 3a). As mentioned, with respect to the apo form, Zn1 and Zn2 positions are only marginally shifted, maintaining a distance of about 4.6 Å between them. The side chain position of key residues involved in the metal atoms coordination, i.e. His116/94, His118/96, Asp120/98, His196/159, Cys221/178 and His263/220, is conserved.

In the binary structures with NDM-1 compound **31** orients similarly to **24,** with which it shares the same sulfhydryl-triazole moiety. Considering the high structural homology along MBLs and the similar chemical structure of inhibitors, a close orientation in the enzyme binding site was expected. With respect to **24** in VIM-2, compound **31** in NDM-1 is further stabilized by hydrophobic interactions formed with Ile28/35, Val67/73 and His263/250, and by a hydrogen bond with Gln119/123 (Fig. 3d). The zinc ions maintain the position they have in the apo form, as well as the coordinating residues, i.e. His116/120, His118/122, Asp120/124, His196/189, Cys221/208, His263/250.

### X-ray crystallography vs modelling binding prediction

Disappointingly, the crystallographic poses of the ligands are quite different from that predicted as the most promising by molecular docking simulations. For compound **24** in VIM-2, the docking prediction orients the molecule in the opposite way, where both zinc ions are coordinated by the sulfhydryl group and stabilized by additional hydrogen bonds with Arg228/185 (Figs. 1c, 3b). Poses more similar to those identified by X-ray analyses were also produced by docking simulations (Fig. 3c) but were not ranked as the best ones. The superposition of the X-ray and of the most similar docking pose for compound **24** is reported in Fig. 3c**.** Still, a slight adjustment in the pose orientation can be observed, in particular because of the presence of Phe61/62, not detected in the X-ray binary complex. The lack of the residue side-chain is likely associated to a higher flexibility and opening of the loop L3 observed in the here reported structure, with respect to the structure used for docking simulations.

For compound **31**, the pose selected during the virtual screening was actually the one most similar to the crystallographic pose (Fig. 3f and S3) but was not the one that received the highest score in silico (Fig. 3e). It should be noted that the amide sidechain of Gln119/123 is flipped in the X-ray binary complex compared to the structure used for docking simulations, allowing it to interact with the ligand pyridyl nitrogen. We can observe a slight shift towards His196/189, possibly because of the flipped Gln119/123 or, alternatively, for the absence of water molecules in docking simulations. As shown, in fact, both compounds are stabilized by a water interacting with one of the triazole nitrogen atoms (Fig. 3a,d). The highest ranking was, instead, assigned to a conformation of compound **31** having the opposite orientation with respect to the crystallographic one (Fig. 3e).

Thus, even if molecular docking guided the selection of the most promising candidates and, indeed, allowed the identification of molecules active towards the selected targets, the predicted orientation, in particular for compound **24**, and the zinc coordination, turned out to be misleading. This failure is likely due to several factors: the flexibility of part of the site that is not taken into account, the lack of waters in docking, and an overestimation of the sulphydryl group capability to coordinate the two zinc ions, with respect to the triazole nitrogens. These findings represent valuable indications to guide any further docking simulation of similar compounds in metallo-based proteins in general and in MBLs binding site in particular^42,43^.

### Identification and characterization of compounds inhibiting BLs of different molecular classes

Most interestingly, some of the compounds were cross-class BLIs, being able to target BLs belonging to different classes (Chart 1). The largest inhibition spectrum was observed for compounds **40** and **46**, located at the centre of the Venn chart. Compound **46**, in particular, inhibits CTX-M-15 at 55%, KPC-2 at 66%, AmpC at 32%, NDM-1 at 79% and VIM-2 at 45%. Compound **40** shows good inhibition towards KPC-2, NDM-1 and AmpC as well (41%, 71% and 50%, respectively). Compound **59** also presents interesting results for CTX-M-15 and KPC-2 (49% and 46%, respectively), and a good inhibition against NDM-1 and VIM-2 (62% and 81%, respectively). The superpositions of the two MBLs and of the three SBLs with compound **59** docked in the binding site are reported in Figs. S11 and S12. The orientation of the compound is well conserved in NDM-1 and VIM-2, reflecting the similarity of the two binding sites. Furthermore, the poses in KPC-2 and CTX-M-15 are also similar, with the exception of the furan ring, which appears to be twisted. A quite different orientation can be observed in AmpC. Interesting compounds are also **24** and **52**, which provided good inhibition against NDM-1 and VIM-2. In particular, compound **24** inhibits NDM-1 at 70% and VIM-2 at 86%, while compound **52** at 80% and 62%, respectively.

For some of the most interesting compounds, those being predicted broad-spectrum or strong MBLs inhibitors, the inhibition constant towards relevant BLs was measured (Table 3). Interestingly, all tested molecules showed inhibition towards MBL enzymes, with *K*_*i*_ values ranging from 60 to 10 μM. Compound **52** demonstrated to be the most active towards NDM-1. Compound **40** also showed, although to a lesser extent, inhibition of CTX-M-15 ESBL enzyme, which ultimately confirms the cross-class inhibition potential of some of the inhibitors described herein and the validity of the in silico screening approach used in this study. Indeed, many identified compounds ultimately proved to be ligands of the predicted targets, either by X-ray crystallography or enzyme assays.

**Table 3:** Inhibition constants of compounds 24, 40 and 52 measured on some representative class A (serine-) and class B (metallo-) β-lactamases.

Compound	β-lactamase	*K*_*i*_ (µM)
**24**	VIM-2	41 ± 4
**40**	CTX-M-15	150 ± 12
	NDM-1	58 ± 9
**52**	NDM-1	10.7 ± 1.4

K_i_ values were determined using the reporter substrate method and a competitive model of inhibition.

### Evaluation of the synergistic activity of the selected compounds

The capability of compound **40**, selected on the basis of its broad-spectrum activity, to potentiate the activity of a β-lactam antibiotic was investigated using recombinant isogenic *E. coli* strains producing the various BLs of the enzyme screen panel, including ESBL CTX-M-15, carbapenemase KPC-2, subclass B1 MBLs VIM2 and NDM-1 and *P. aeruginosa* AmpC (class C) (Table 4). The compound did not, expectedly, show any direct antibacterial activity, but was able to potentiate the activity of ampicillin, depending on the produced β-lactamase type. The susceptibility of the strains producing CTX-M-15 or VIM-2 was significantly increased in the presence of **40**, while a more modest effect was observed with strains producing KPC-2 or NDM-1. No effect was observed on the strain producing an AmpC-type enzyme (PDC-1). It should be noted that the contribution of these different enzymes to ampicillin resistance also largely varies.

**Table 4:** In vitro antibacterial synergistic activity of compound 40 on recombinant isogenic *E. coli* strains evaluated by the disk diffusion method (See Materials and Methods for details).

Plasmid carried by the *E. coli* DH5α strain	Diameter of growth inhibition zone (mm)				
	Ampicillin (10 µg)	Ampicillin (10 µg) + 40 (80 µg)	Ampicillin (10 µg) + avibactam (4 µg)	Ampicillin (10 µg) + EDTA (70 µg)	40 (80 µg)
pLBII-CTX-M-15	≤ 6	16	26	–	≤ 6
pLBII-KPC-2	9	11	22	–	≤ 6
pLBII-AmpC_*P.*_* aeruginosa*	19	19	25	–	≤ 6
pLBII-VIM-2	9	17	–	24	≤ 6
pLBII-NDM-1	16	18	–	21	≤ 6

DH5α strain was transformed with a derivative of the pLB-II vector, in which the β-lactamase gene was cloned)^44^. Avibactam and EDTA were used as inhibition controls for SBLs and MBLs, respectively.

– not determined.

Based on these encouraging results, the synergistic activity of **40** and other compounds was tested on clinical isolates producing different types of β-lactamases, including a plasmid-encoded class C enzyme CMY-2, KPC-3 serine-carbapenemase, VIM-1 and NDM-1 metallo-carbapenemase (Table 5). Interestingly, compound **40** was able to reduce the MIC of imipenem from 16 to 1 μg/mL in an NDM-1 producing clinical isolate, being below the resistance breakpoint as defined by both CLSI and EUCAST. The potentiation effect of this compound was lower in other strains. For the other tested compounds, and in apparent contrast with their inhibition profile, no significant MIC reduction was observed, indicating that such compounds do not likely have the potential to reach the periplasmic space. However, it is not clear at this stage whether this would rely on the compound specific physical–chemical properties or whether the compounds could be substrate of efflux pumps, commonly produced by MDR clinical isolates.

**Table 5:** In vitro antibacterial activity of imipenem (IPM), ceftazidime (CAZ) and cefepime (FEP), in the absence and presence of 32 µg/mL of tested compound, on clinical isolates producing several types of clinically relevant β-lactamases.

Compound (32 μg/mL)	MIC (μg/mL)				
	*K. pneumonia* SI-109 $\left( {bla_{{\text{KPC } - \text{ 3}}}}^{+} \right)$	*K. pneumonia* SI-001G $\left( {bla_{{\text{VIM } - \text{ 1}}}}^{ + } \right)$	*E. Coli* SI-002M $\left( {bla_{{\text{NDM } - \text{ 1}}}}^{ + } \right)$	*E. Coli* SI-2602P $\left( {bla_{{\text{CMY } - \text{ 2}}}}^{ + } \right)$	
	IPM	IPM	IPM	CAZ	FEP
None	16	8	16	128	2
10	16	8	16	128	2
20	16	8	16	128	2
24	16	8	16	128	2
31	16	8	16	128	2
32	16	8	16	128	2
40	16	4	1	128	1
46	16	8	16	128	2
52	16	8	8	128	1
55	16	8	16	128	2
59	16	8	16	128	1

## Conclusions

Herein, we have presented the parallel screening that led to a library of 66 validated candidates targeting BLs: class A and C SBLs (CTX-M-15, KPC-2, AmpC) and class B1 MBLs (NDM-1 and VIM-2). Overall, the VS campaign, performed with the ad hoc developed version of the molecular docking software FLAPdock, gave quite good results, with a high percentage of active molecules being found: ten compounds for CTX-M-15, twenty-two for KPC-2, thirty-one for NDM-1 and ten for VIM-2. Moreover, two compounds were found active against AmpC.

Specific scaffolds were identified as being promising for each class. For class B1 NDM-1, the active compounds often present a thiol, a thiosemicarbazide or thiosemicarbazone moiety or, in general, electron pair donors. For VIM-2, presenting more stringent structural requirements, triazole-based ligands appeared to be the most efficient. Despite the slightly different binding site architectures, several compounds were found to be able to concomitantly inhibit both enzymes. Interestingly, the most active molecules present a triazole-thiol moiety.

Good results were also found for SBLs and, in particular, for class A KPC-2. This enzyme, just like NDM-1 and VIM-2, requires the presence of compounds having electron pairs that can interact with the catalytic serine and with the other H-bond donor residues lining the pocket. The highest inhibition percentage was found for sulfonamide and tetrazole-based derivatives. Fewer promising results were obtained for class A CTX-M-15 and class C AmpC and no specific scaffold was identified. Two X-ray crystallographic complexes were solved at high resolution with NDM-1 and VIM-2, deciphering the binding requirements for MBLs inhibition, and providing valuable insights to be considered in molecular docking simulations when metallo-proteins are involved.

Interestingly, many compounds were found to inhibit BLs belonging to same class and a few to inhibit BLs belonging to different classes. More in detail, three compounds (**40**, **46** and **59**) target all considered BLs, resulting broad-spectrum inhibitors. Furthermore, compound **40** inhibited both CTX-M-15 and NDM-1 BLs with *K*_*i*_ values of ≈100 μM and showed a significant 16-fold potentiation of imipenem activity on a NDM-1-producing *E. coli* clinical isolate, with a resulting MIC value in the susceptibility range. Although this was achieved at a rather high concentration of inhibitor (32 μg/mL), these data allowed to identify **40** as a promising starting point for the design of completely novel BL inhibitors, with demonstrated in vitro activity in whole cell assays.

Inhibitors sharing similar chemical structures and with comparable inhibition activity have been already reported by ourselves^34,35,45^, and by other groups^46,47^. However, with respect to the here presented compounds, the molecules so far proposed in literature were designed to act only towards MBLs. To our knowledge, this is one of the first computational studies that succeeded in the identification of broad-spectrum inhibitors active towards BLs belonging to different classes. The difficulty of designing and developing non-covalent inhibitors that can target SBLs, the worrisome spread of MBLs and, most importantly, the co-production of carbapenemases belonging to different classes, highlight the significance of the obtained results. The development of ligands acting as broad-spectrum inhibitors would represent a significant step forward for the containment of antibiotic resistance.

With respect to recently approved molecules with broad spectrum profile and nanomolar affinity^12^, the here identified inhibitors, despite their low micromolar activity, share a cross-class inhibition and represent novel drug-like leads with valuable biological activity to be directed to hit-to-lead development.

## Materials and methods

### Molecular modelling

#### MBL numbering

For clarity, when referring to MBL residues, both the consensus and the PDB numbering will be given in the text (consensus/PDB)^48^. In the figures, only the consensus numbering scheme has been adopted. For class C AmpC we adopted the SANC (Standard Alignment-based Numbering) standard numbering scheme^49^.

#### Protein modelling

Protein X-ray structures were retrieved from the Protein Data Bank, and chosen according to the crystallographic resolution, the absence of mutations and the presence/absence of complexed ligands, in order to better evaluate the intrinsic dynamics of the protein matrix and local binding site flexibility. When relevant ligand-induced binding-site rearrangement (induced-fit effect) was observed, more than one X-ray structure was used for the same protein. Co-crystallized ligands, along with water molecules, were removed. Each structure was carefully inspected using the *flapsite* algorithm (implemented in FLAP, developed and licensed by Molecular Discovery Ltd.) to automatically detect the binding site pocket where the compounds would be docked.

CTX-M-15 was considered in both the apo and holo form in complex with avibactam (PDB codes 4hbu and 4hbt, respectively), KPC-2 in the apo form (PDB code 3dw0), NDM-1 in the apo and holo form in complex with ampicillin (PDB codes 3spu and 5zge, respectively), VIM-2 in complex with a mercaptocarboxylate inhibitor (PDB codes 2yz3). Finally, the complex with 4-carboxyphenilboronic acid was used for AmpC (PDB code 1kdw).

#### Database treatment

The Specs database (www.specs.net) was screened against the selected β-lactamases. The original library of about 300,000 compounds was filtered according to molecule logP, as predicted by MoKa^39^. Only compounds with logP values lower than 3 (thus likely to be soluble for the subsequent in vitro analyses) were retained. Tautomers and protomers were built using MoKa. Only tautomers and protomers with a predicted percentage higher than 10 were retained. The final library contained about 27,000 compounds.

#### Virtual screening

Structure-based virtual screening (SBVS) analyses were performed using FLAPdock implemented in FLAP (Fingerprint for Ligands and Proteins), developed and licensed by Molecular Discovery Ltd^50^. FLAP is based on GRID Molecular Interaction Fields (MIFs), which describe both ligands and protein active sites, and can be used to find complementary ligands with a receptor-based pharmacophore approach^38,51^.

#### The FLAPdock fragment-based approach

FLAPdock, recently published and described in^38^, is a fragment-based docking approach. Ligands are split into fragments and each fragment is first scored using a weighted sum of the similarity between the GRID MIFs (shape, H-bond donor, H-bond acceptor, hydrophobicity) that are calculated for the active site and the pseudo-MIFs calculated for the fragment. A more accurate scoring function (FLAP S-Score), which includes both Lennard–Jones and dielectric corrected Coulombic energetic terms, is then applied. The best ranked solutions are retained and the fragment anchor point used to incrementally construct the ligand, while various torsion angles for each added fragment are explored at each stage, retaining the best ranked solutions for the next fragment addition. For sites containing metals, any known metal-interacting groups in the ligand are biased towards interacting with the metal ion via their charge interaction and, in the specific case of MBLs, sulfhydryl compounds are considered deprotonated because of the zinc effect, which lowers the p*K*_a_ of bound thiol groups by about two orders of magnitude^32,33^.

The library was first docked in each of the selected BL binding site with lower accuracy in order to speed up the calculations. The best 1% ranked molecules of the library were then re-docked using the higher accuracy modality, which takes more computational time, but provides more accurate predictions.

#### Compound selection

The compounds docked within the protein binding site were visually inspected. The most interesting molecules, according to the FLAP S-Score, the number of hydrogen bonds formed with the pocket surrounding residues and the chemical diversity, were purchased and tested in inhibition assays. In some cases, the mentioned criteria indicated that a compound was promising for more than one target. This happened more often for NDM-1 and VIM-2, which belong to the same class and share a relatively high similarity at the binding site level (see the Results section for further details). These compounds have a higher chance of being broad-spectrum and were prioritized during selection.

The 66 selected molecules were purchased and experimentally tested for inhibition towards CTX-M-15, KPC-2, AmpC, NDM-1 and VIM-2 (Tables S1–S3).

### Protein production, purification and inhibition assays

All proteins employed in the in vitro validation were produced and purified as already reported^38^. For proteins used in the crystallographic studies the following protocols were employed.

#### Cloning, production and purification of recombinant VIM-2

BlaVIM-2 gene lacking signal peptide (26–266, UniProtKB—Q9K2N0) was purchased by Eurofins Genomics, and directly cloned into pETite N-His SUMO Kan Vector (Lucigen). Overexpression of the N-His SUMO VIM-2 protein was performed transforming *E. coli* Lemo21(DE3) cells (NEB) and grown in LB medium (25 mg/L kanamycin, 17 mg/L chloramphenicol) at 37 °C. Large scale expression was induced at 0.5 OD600 with 0.2 mM Isopropyl-β-d-1thiogalactopyranoside (IPTG), overnight at 25 °C. Cell pellet was resuspended in lysis buffer (20 mM Tris–HCl pH 8.0, 150 mM NaCl, 1 mM ZnCl2, 10% glycerol and 20 mM imidazole) supplemented with protease inhibitors cocktail (Roche), and lysed by French press. N-His SUMO VIM-2 was purified from soluble fractions through IMAC (HisTrap HP 1 mL column, GE Healthcare) and eluted by 0.5 M imidazole. Fractions containing N-His SUMO VIM-2 were buffer exchanged with PD10 desalting columns (GE Healthcare) in 20 mM Tris–HCl pH 7.5, 150 mM NaCl and 10% glycerol. N-His SUMO removal was performed through cleavage of the purified protein using SUMO express Protease (Lucigen). SUMO express protease and uncleaved N-His SUMO VIM-2 were removed by IMAC.

#### Cloning Expression and purification of recombinant NDM-1

NDM-1 protein without signal peptide (28–270, UniProtKB—C7C422) was produced and purified as described before^52^.

All the enzymes used in this study were further purified by size exclusion chromatography (Superdex 200 Hiload 16/60, GE Healthcare) and stored at 193 K for crystallization purposes. Optimized buffers were used for each of them: 20 mM Hepes, 100 mM NaCl pH 7.0 for NDM-1; 30 mM Hepes, 150 mM NaCl pH 7.1 for VIM-2.

#### Enzyme inhibition assays

The entire library of 66 purchased compounds was screened, without further purification, against the selected β-lactamase panel using a colorimetric assay and a 96-well multiplate reader (*Spectramax-190*-*Molecular Devices*). Each compound was dissolved in DMSO in 10 mM stock solutions. Inhibition activity assays were performed in the reaction buffer, which consisted of 50 mM PB + 50 mM KCl, at pH 7.0 and 25 °C with 0.01% v/v Triton x-100, to avoid compound aggregation and promiscuous inhibition, and using CENTA 250 µM as the reporter substrate^53,54^. Reactions started with the addition of the enzyme and were monitored at 405 nm, over a time course of 300 s. Inhibition activity was tested at a fixed ligand concentration of 200 µM, and three replicates were performed for each compound. Standard error was below 10%. Inhibition constants were determined using a competitive inhibition model and by measuring the initial reaction rate of hydrolysis of a reporter substrate (150 μM imipenem, for VIM-2 and NDM-1 enzymes, or 100 μM cephalothin for CTX-M-15) in 50 mM HEPES buffer (pH 7.5), by means of spectrophotometric assays in the absence and presence of a variable inhibitor concentration, as previously described^55^. Reaction rates were measured in triplicates.

### X-ray crystallography

Purified truncated NDM-1 β-lactamase was crystallized according to previously determined conditions^52^, while VIM-2 was submitted to crystallization trials according to well established protocols proved to be successful with other ligands^56^. Briefly, for NDM-1, a purified protein sample was concentrated to 50 mg/mL and submitted to an isothermal vapor diffusion crystallization setup (0.8 μl total volume drops, 1:1 protein-precipitant ratio, 293 K). NDM-1 in complex with compounds **31** was obtained only in a two-phase co-crystallization setup, according to the procedure recently proposed by Leiros and colleagues^56^, while all the other conditions and compounds tested failed to give crystals of NDM-1 in complex with any compounds. The same two-phase strategy proved to be successful for both NDM-1 and VIM-2. VIM-2, concentrated to 10 mg/mL, was crystallized as previously described^56,57^. Briefly, VIM-2 supplemented with 0.5 mM ZnCl_2_ was crystallized in isothermal vapor diffusion conditions (0.8 μL total volume drops, 1:1 protein-precipitant ratio, 293 K), using as precipitant buffer 0.25 M magnesium formate, 27% PEG3350. Diffracting crystals of VIM-2 in complex with **24** were obtained with hand-made fresh crystallization trials on top of dried **24** compound, as indicated above.

In all cases, crystals were cryo-protected with 20% ethylene glycol v/v added to the precipitant solution and flash frozen in liquid nitrogen. Diffraction data were collected at the Swiss Light Source (SLS) at the Paul Scherrer Institut in Villigen, Switzerland. Both NDM-1 and VIM-2 crystals gave complete datasets and diffracted to resolutions better than 1.5 Å.

The structures were solved through molecular replacement by Phaser software^58^, using ccp4i2 interface^59^. The protein models 6Q2Y for NDM-1 and 4BZ3 for VIM-2 were used as starting template^57^. Refinement was carried out by Refmac5^60^, refine (Phenix interface)^61^ and manual adjustment through the graphic software Coot^62^. The identification of compounds bound to the MBLs active sites was performed through a detailed inspection of Fourier Difference maps. Ligand molecules were built and optimized by Elbow (Phenix)^63^, and then fitted in the maps using Coot tools. Their position was adjusted by local refinement and further steps of global refinement. Completion of the models was achieved by water molecules fitting and other ligands identification, as metal ions and buffer molecules. In particular, the analysis of both NDM-1 and VIM-2 crystals anomalous scattering maps confirmed the presence of the two conserved and catalytically relevant zinc ions in the active sites (data not shown). Likewise, for NDM-1 crystals we identified calcium ions coordinated by acidic residues nearby the catalytic site, as already described^52^, while for VIM-2 we detected additional zinc ions coordinated by formate molecules at the interface, with symmetry mates in the crystal packing. PyMOL Molecular Graphics software and Phenix tool for omit maps calculations were used to generate illustrations.

### In vitro antibacterial susceptibility assays

The potential of the described molecules to increase the activity of a β-lactam antibiotic was investigated using recombinant isogenic *E. coli* strains producing different types of β-lactamases, including the class A ESBL CTX-M-15 and the KPC-2 carbapenemase, the subclass B1 MBLs VIM-2 and NDM-1 and the class C *P. aeruginosa* AmpC (PDC-1) enzyme. The β-lactamase genes were cloned in the pLB-II vector as previously described^44^, and the resulting plasmid used to transform *E. coli* DH5α. A disk diffusion method (known as combo test) was used (considering its lower requirements in terms of compound quantity) to evaluate the synergistic activity of the compounds, as previously described^27^. Briefly, the diameter of the growth inhibition zone of an ampicillin-containing disk (10 µg, Oxoid, Milan, Italy), on which either the tested compounds (80 µg), EDTA (MBL inhibition control, 70 µg) or avibactam (SBL inhibition control, 4 µg) were dispensed (max. volume, 15 μl), were compared to that obtained with the antibiotic disk control. Results were read after aerobic incubation at 35 ± 1 °C for 18–24 h.

Alternatively, the broth microdilution method was used. In this case, clinical isolates producing a plasmid-encoded class C enzyme CMY-2, a KPC-3 serine-carbapenemase, VIM-1 or NDM-1 metallo-carbapenemase were used^64^. Strains were in our antibiotic-resistant clinical isolates collection and were recovered from various Italian hospitals. Minimal Inhibitory Concentrations (MICs) of imipenem, ceftazidime and cefepime were determined at least twice by the broth microdilution method in Mueller–Hinton broth, according to the Clinical Laboratory Standards Institute guidelines^65^, in the absence and presence of 32 μg/mL of the tested compound. Plates were incubated aerobically at 35 ± 1 °C for 18–24 h before reading.

## Supplementary information

Supplementary information

## Data Availability

Atomic coordinates and structures factors have been deposited in The Protein Data Bank for immediate release as PDB ID: 6tgi (VIM-2 in complex with compound 24) and 6tgd (NDM-1 in complex with compound 31).
