# Supplementary material for: Virtual screening identifies broad-spectrum β-lactamase inhibitors with activity on clinically relevant serine- and metallo-carbapenemases
Source: Sci Rep. 2020 Jul 29;10:12763. doi: 10.1038/s41598-020-69431-y (PMC7391774; doi:10.1038/s41598-020-69431-y)
Supplement: Supplementary file 1 — Supplementary information [file 41598_2020_69431_MOESM1_ESM.pdf]

Virtual screening identifies broad-spectrum β-lactamase inhibitors with activity on clinically relevant serine- and metallo-carbapenemases

Francesca Spyrakis[1,†,‡], Matteo Santucci[1,‡], Lorenzo Maso[2,‡], Simon Cross[3], Eleonora Gianquinto[4], Filomena Sannio[5], Federica Verdirosa[5], Filomena De Luca[5], Jean-Denis Docquier[5], Laura Cendron[2], Donatella Tondi[1], Alberto Venturelli[6], Gabriele Cruciani[7*], Maria Paola Costi[1*]

[1] Department of Life Sciences, University of Modena and Reggio Emilia, Via Campi 103, 41125, Modena, Italy; [2] Department of Biology, University of Padua, Viale G. Colombo 3, 35121, Padua, Italy; [3] Molecular Discovery Limited, Unit 501 Centennial Park, Centennial Avenue, Borehamwood, Hertfordshire, WD6 3FG, United Kingdom; [4] Department of Drug Science and Technology, University of Turin, Via Pietro Giuria 9, 10125, Turin, Italy; [5] Department of Medical Biotechnology, University of Siena, Viale Bracci 16, 53100, Siena, Italy; [6] TYDOCK PHARMA S.r.l., Strada Gherbella 294/b, Modena, 41126, Italy; [7] Department of Chemistry, Biology and Biotechnology, University of Perugia, Via Elce di Sotto 8, 06123, Perugia, Italy

‡ These authors equally contributed to the work.
† Current address: Department of Drug Science and Technology, University of Turin, Via Pietro Giuria 9, 10125, Turin, italy

*** Correspondence:**

Maria Paola Costi

Department of Life Sciences, University of Modena and Reggio Emilia, Via Campi 103, 41125, Modena, Italy.
phone: 0039 059 2058579
e-mail: mariapaola.costi@unimore.it

Gabriele Cruciani

Department of Chemistry, Biology and Biotechnology, University of Perugia, Via Elce di Sotto 8, 06123, Perugia, Italy.
phone: 0039 075 5855629
e-mail: gabri@chemiome.chm.unipg.it

Table of contents

Table S1. Compound library structures and properties……………………………………….…………..S3

Table S2. Specs codes of the tested compounds…………………………………………..……...S7

Figure S1. Overall fold and detailed view of the active site of class A and class B β-lactamases ……………………………………………………………………………………………...…S8

Figure S2. Docking pose of compound 52 in NDM-1 active site……………..…………..……...S9

Figure S3. Docking pose of compound 31 in VIM-2 active site……………..…………..……..S10

Figure S4. Docking poses of compound 24 in NDM-1 and VIM-2 active sites………….....…...S11

Figure S5. Docking poses of compound 54 in NDM-1 and VIM-2 active sites……………….....S12

Figure S6. Docking pose of compound 9 in KPC-2 active site...…………………….……...….S14

Figure S7. Docking pose of compound 7 in CTX-M-15 active site…………….……..…..…….S15

Figure S8. Docking pose of compound 54 in AmpC active site……………...………......…...…S16

Figure S9. Superimposition of NDM-1 (cyan) and VIM-2 (grey) active sites…….…………...S17

Figure S10. Superimposition of KPC-2 and CTX-M-15 binding site…………………………S18

Figure S11. Docking pose of compound 59 in NDM-1, VIM-2, KPC-2 and CTX-M-15………………………………………………………………………………………………..S19

Figure S12. Superposition of NDM-1, VIM-2, KPC-2, CTX-M-15, KPC-2 and AmpC binding to compound 59……………………………………………………………………………………...S20

Table S4. Data collection and refinement statistics for the VIM-2-compound 24 binary complex……………………………………………………………………………………………S21

Table S5. Data collection and refinement statistics for the NDM-1-compound 31 binary complex……………………………………………………………………………………………S22

Scheme S1. ΔVIM-2 synthetic gene sequence………………………………………………..…S23

Table S1. Compound library structures and properties.

ID	STRUCTURE	MW	LogP	ID	STRUCTURE	MW	LogP
**1**	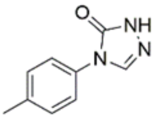	175.19	0.81	**2**	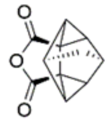	188.18	2.34
**3**	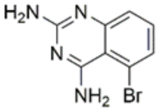	239.07	2.04	**4**	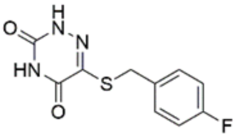	253.26	1.69
**5**	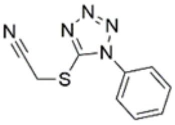	217.25	0.35	**6**	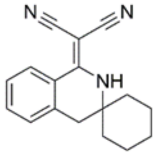	263.34	3.16
**7**	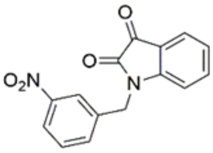	298.75	2.36	**8**	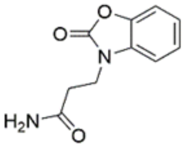	206.2	0.19
**9**	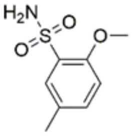	201.25	1	**10**	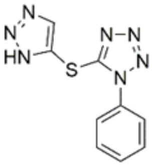	245.27	0.48
**11**	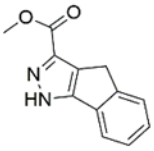	214.22	2.15	**12**	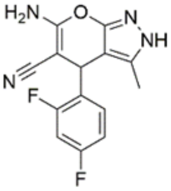	288.26	1.55
**13**	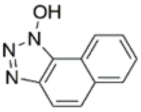	185.19	1.29	**14**	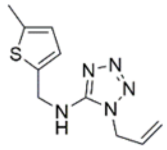	235.31	1.78
**15**	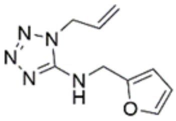	205.22	0.78	**16**	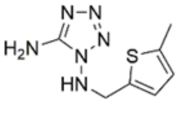	210.26	-0.23
**17**	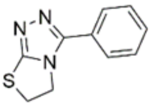	203.27	2.58	**18**	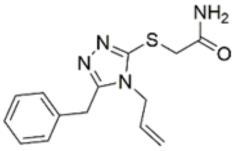	288.37	2.03

**19**	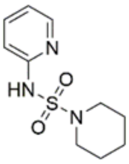	241.31	1.32	**20**	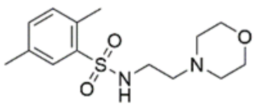	298.41	1.24
**21**	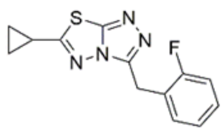	274.32	3.78	**22**	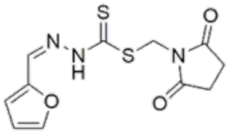	297.36	1.1
**23**	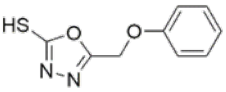	208.24	1.41	**24**	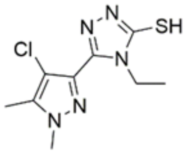	257.75	2.54
**25**	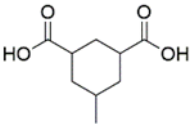	186.21	1.37	**26**	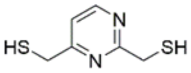	172.28	0.92
**27**	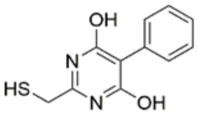	234.28	2.73	**28**	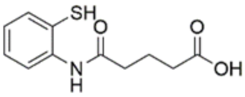	239.3	1.47
**29**	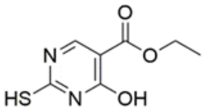	200.22	2.48	**30**	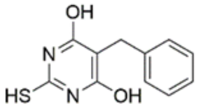	234.28	1.32
**31**	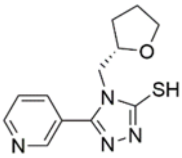	262.34	1.58	**32**	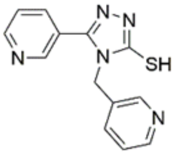	269.33	1.57
**33**	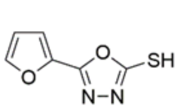	168.18	0.76	**34**	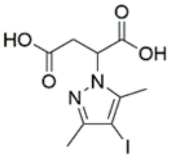	338.1	1.21
**35**	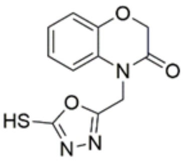	263.28	-0.26	**36**	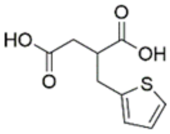	214.24	1.19
**37**	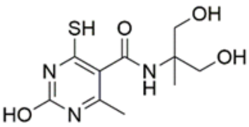	273.31	0.2	**38**	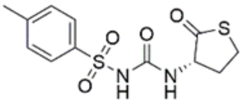	314.38	0.63

**39**	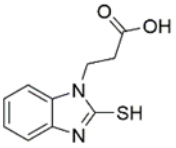	222.27	2.22	**40**	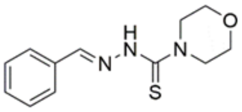	249.34	2.27
**41**	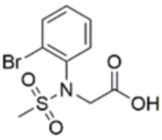	308.15	1.88	**42**	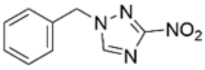	204.19	1.31
**43**	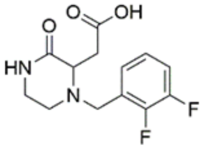	284.26	0.58	**44**	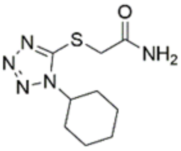	241.32	0.38
**45**	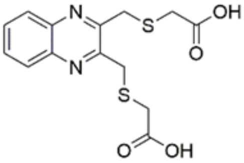	338.41	0.31	**46**	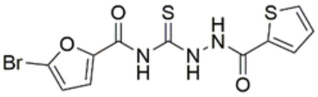	374.24	2.52
**47**	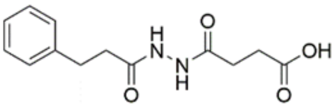	264.28	0.36	**48**	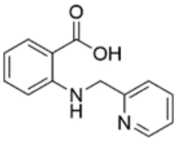	228.25	2.74
**49**	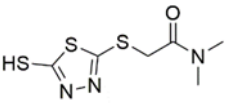	235.36	-0.38	**50**	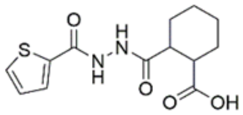	296.35	1.59
**51**	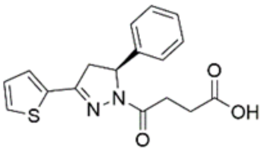	328.39	3.34	**52**	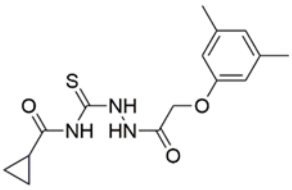	321.4	2.51
**53**	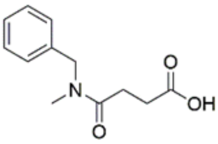	221.26	0.97	**54**	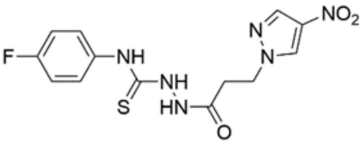	352.35	0.81
**55**	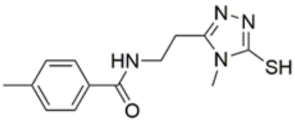	276.36	2.44	**56**	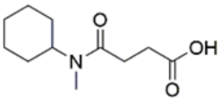	213.28	1.53

**57**	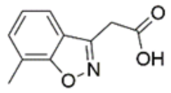	191.19	1.7	**58**	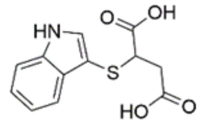	265.29	1
**59**	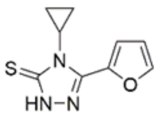	207.26	2.41	**60**	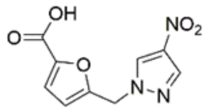	237.17	1.13
**61**	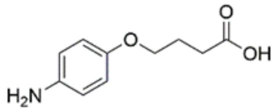	195.22	1.39	**62**	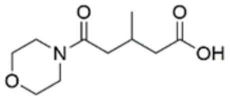	215.25	-0.22
**63**	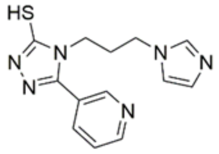	286.36	1.81	**64**	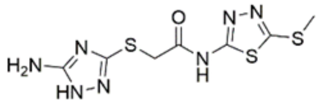	303.39	0.15
**65**	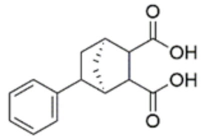	260.29	2.47	**66**	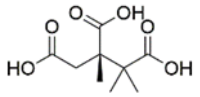	218.21	0.24

Table S2. Specs codes of the tested compounds.

ID	SPECS IDENTIFIER	ID	SPECS IDENTIFIER	ID	SPECS IDENTIFIER
1	AA-516/30041002	23	AG-690/37079058	45	AI-204/31689049
2	AB-131/42302812	24	AK-968/41170774	46	AN-329/41559355
3	AE-413/20011015	25	AE-562/12222692	47	AN-329/40149576
4	AF-399/12447467	26	AC-907/34118003	48	AN-465/43369604
5	AG-205/06470054	27	AC-907/34129008	49	AG-205/15155100
6	AG-687/36202018	28	AK-918/37196002	50	AN-329/41402637
7	AG-690/09906022	29	AG-690/11383008	51	AP-845/42065405
8	AG-690/11081006	30	AG-205/12908115	52	AN-329/42100883
9	AH-487/42822072	31	AO-365/43328944	53	AM-814/41091290
10	AI-204/31688006	32	AO-365/43402945	54	AK-968/41924915
11	AI-204/33265065	33	AH-034/11364396	55	AR-196/42792362
12	AK-777/10808003	34	AG-205/13184001	56	AN-329/42613040
13	AK-830/13217050	35	AK-820/13219002	57	AP-501/43243739
14	AN-465/43411406	36	AI-942/13331196	58	AI-204/42879130
15	AN-465/43411432	37	AJ-430/34386003	59	AP-853/43405475
16	AN-465/43411492	38	AI-204/31691001	60	AK-968/15363563
17	AO-365/43401568	39	AQ-086/43467808	61	AQ-086/43410896
18	AO-476/43417681	40	AF-399/15030141	62	AS-662/43412853
19	AP-124/43382925	41	AG-690/15438978	63	AO-365/43402938
20	AP-263/43417957	42	AK-968/14004674	64	AP-853/43416208
21	AP-501/43286831	43	AN-329/43219379	65	AK-918/43446365
22	AA-504/32628033	44	AN-023/12770009	66	AE-562/12245652

Figure S1. Overall fold (left) and detailed view of the active site (right) of class A (a, KPC-2, PDB code 5ul8) and class B (b, NDM-1, PDB code 5zge) β-lactamases. Residues are numbered according to the BBL consensus numbering scheme.

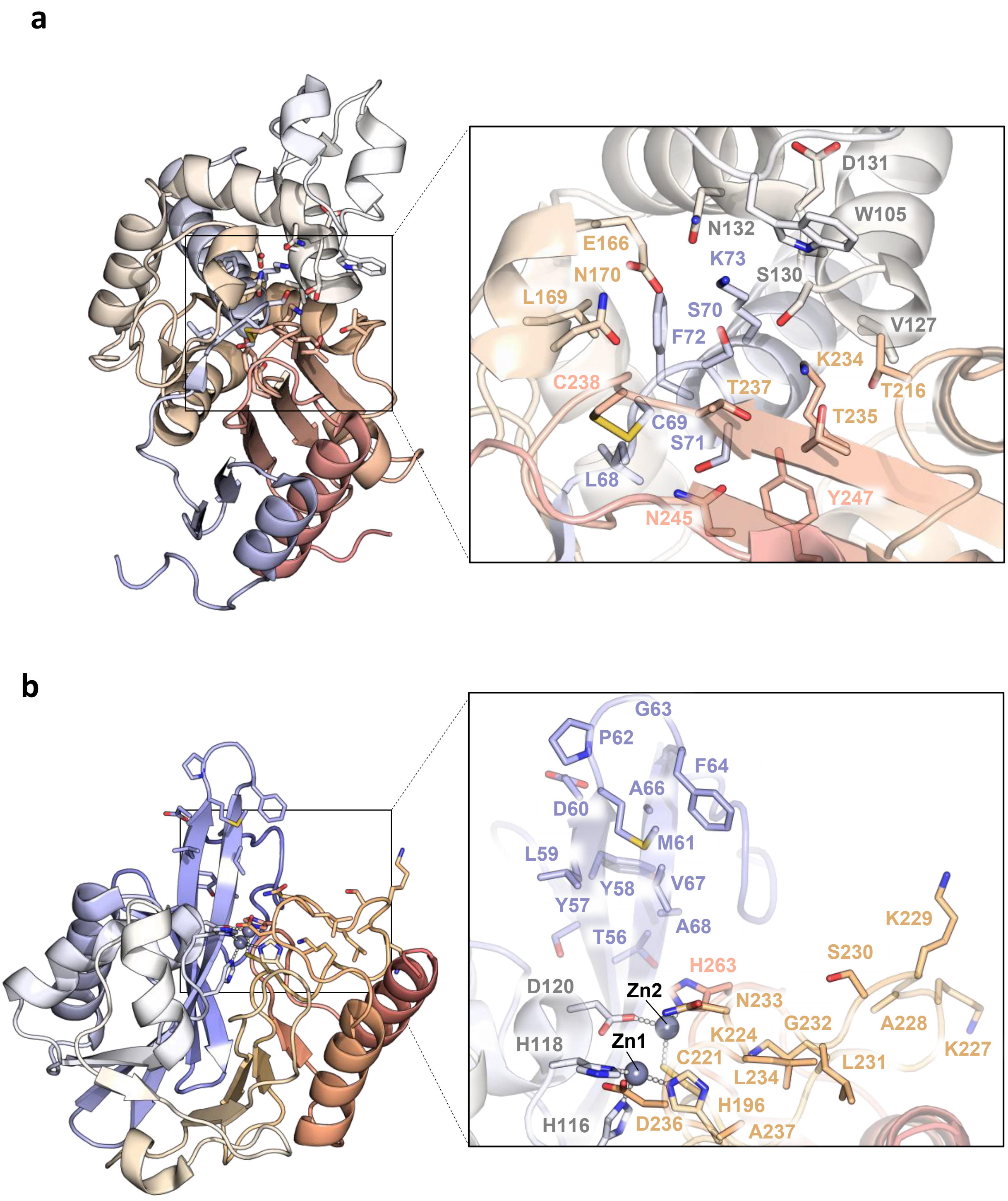

Figure S2. Docking pose of compound **52** in the NDM-1 active site. The hydrophobic and H-bond acceptor Molecular Interaction Fields are represented as yellow and red contours, respectively. Residues are numbered according to the BBL consensus numbering scheme.

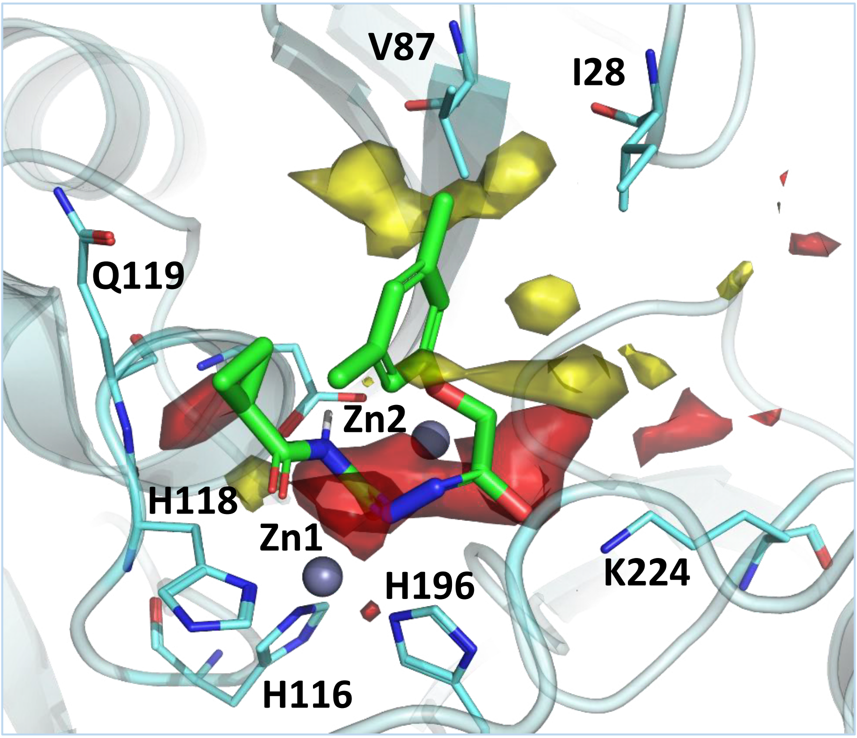

Figure S3. Docking pose of compound **31** in the VIM-2 active site (**a**) (PDB code 2yz3) and corresponding Molecular Interaction Fields (**b**). The protein is shown as cartoons, the compound is shown as capped sticks, the residues lining the pocket in wireframe and H-bonds as dashed lines. The hydrophobic and H-bond acceptor Molecular Interaction Fields are represented as yellow and red contours, respectively. Residues are numbered according to the BBL consensus numbering scheme.

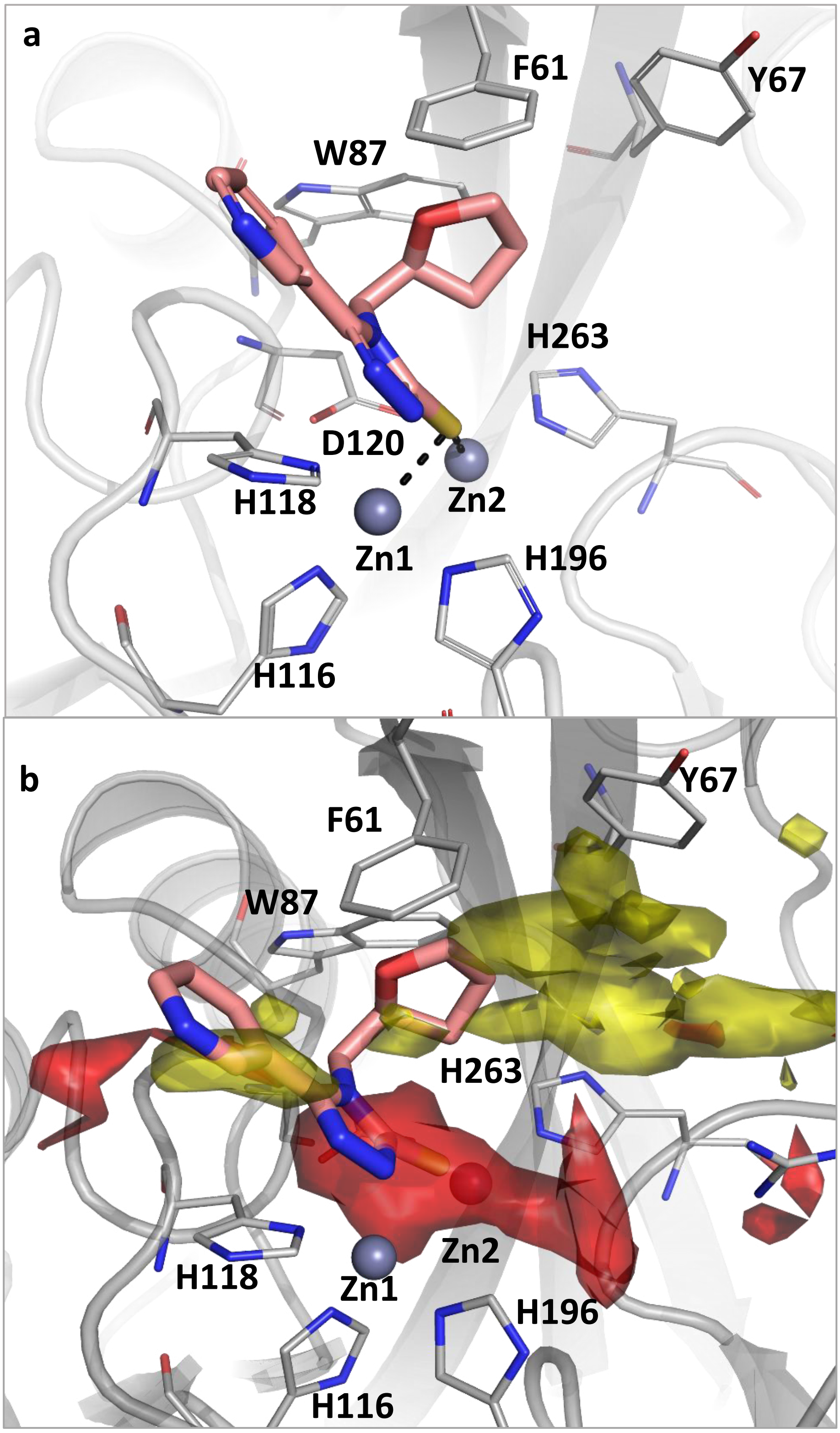

Figure S4. Docking poses of compound **24** in the NDM-1 (**a**) and VIM-2 (**b**) active sites. The hydrophobic and H-bond acceptor Molecular Interaction Fields are represented as yellow and red contours, respectively. Residues are numbered according to the BBL consensus numbering scheme.

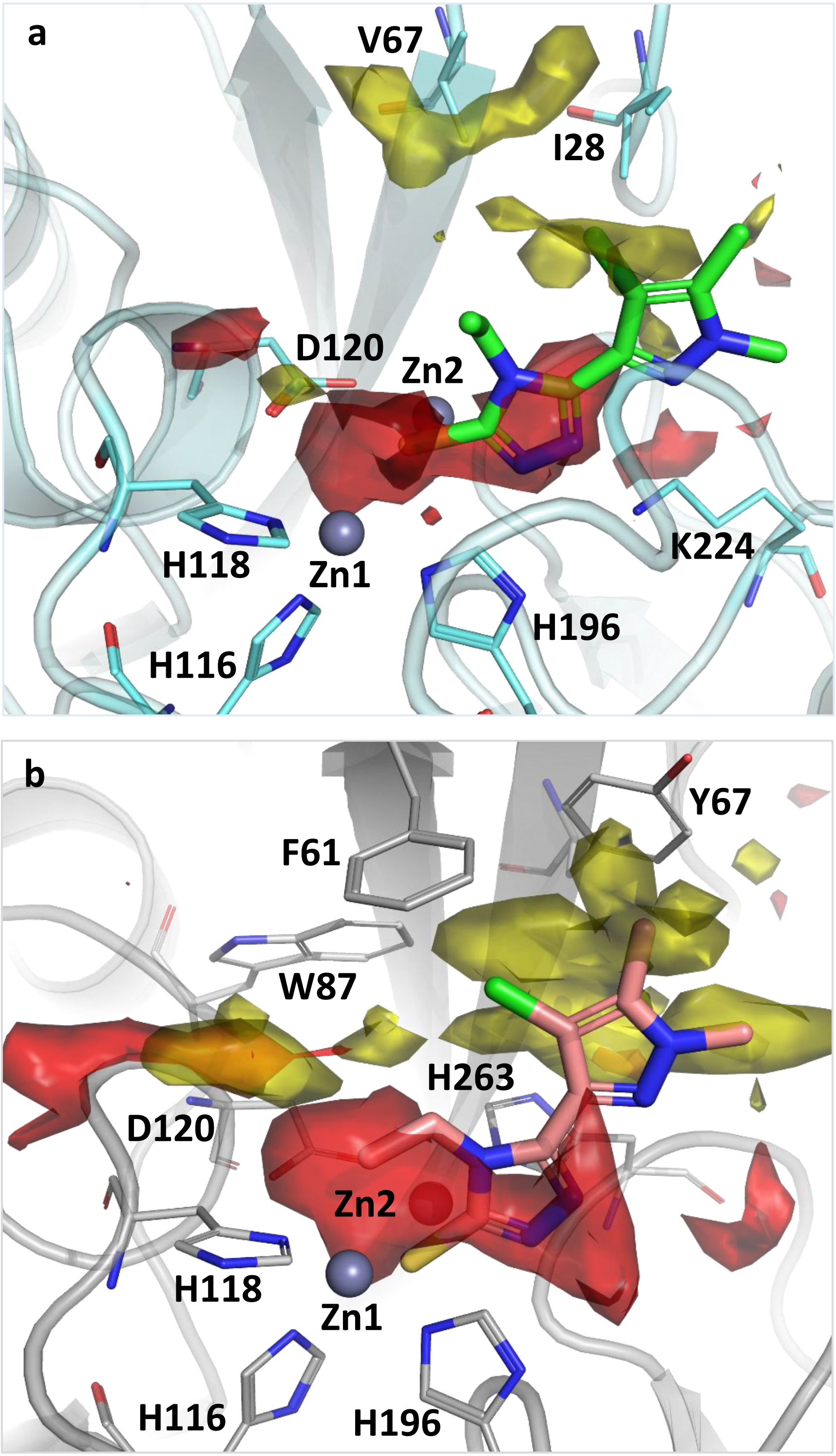

Figure S5. Docking poses of compound **54** in the NDM-1 (**a**) and VIM-2 (**b**) active sites (PDB codes 3q6x and 2yz3, respectively) and corresponding Molecular Interaction Fields (**c, d**). Proteins are shown as cartoons, the compounds are shown as capped sticks, the residues lining the pocket in wireframe and H-bonds as dashed lines. The hydrophobic and H-bond acceptor MIFs are represented as yellow and red contours, respectively. Residues are numbered according to the BBL consensus numbering scheme.

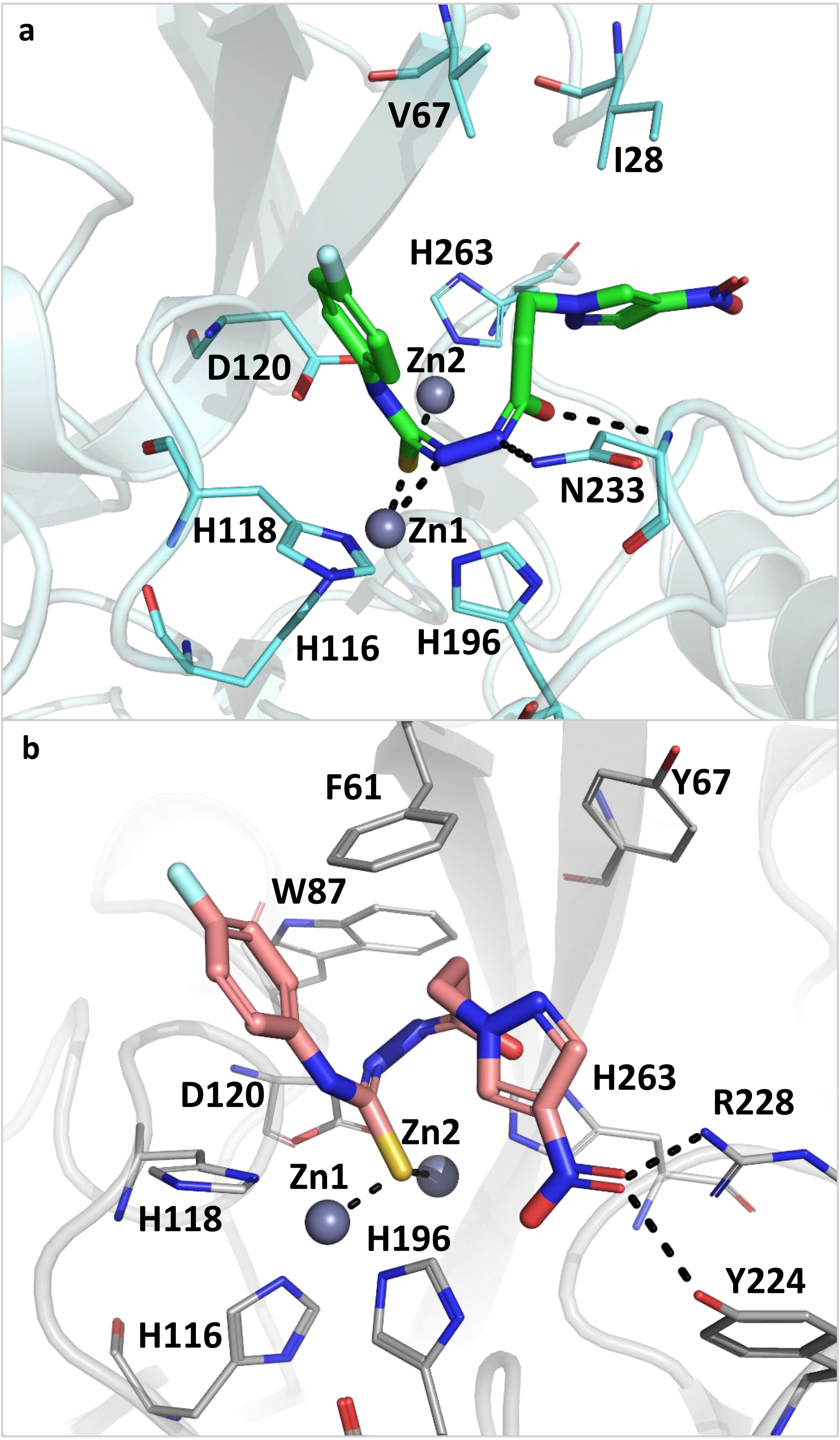

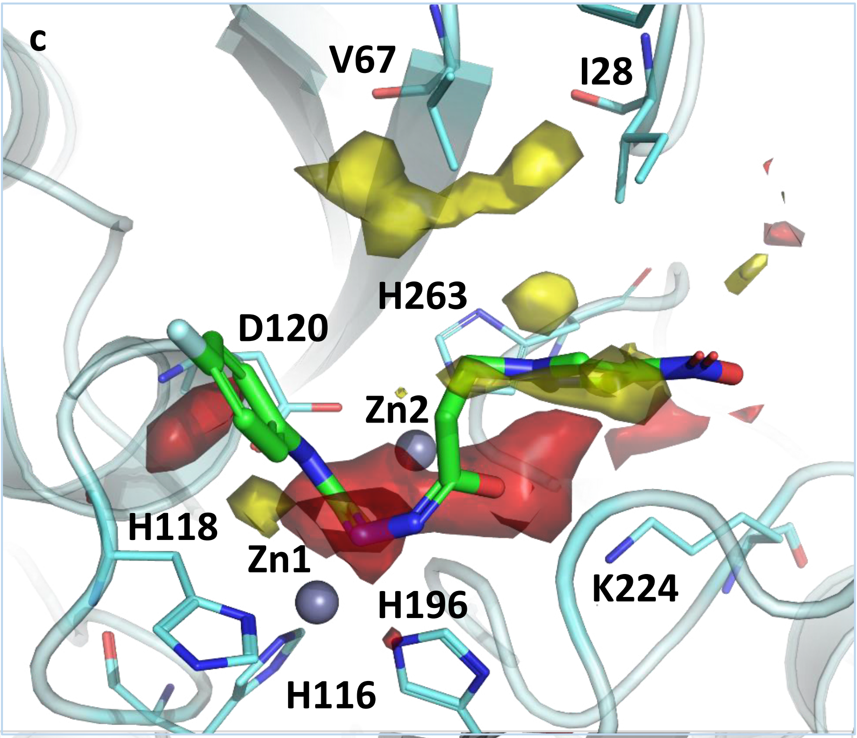

c
V67
I28
H263
D120
Zn2
H118
Zn1
H196
K224
H116

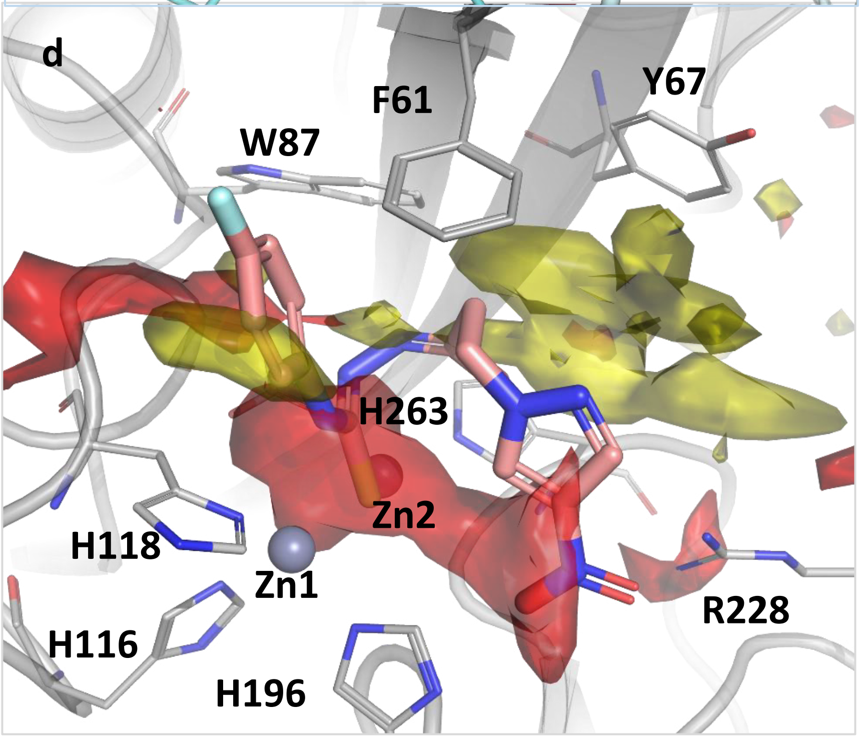

d
F61
Y67
W87
H263
Zn2
H118
Zn1
R228
H116
H196

Figure S6. Docking pose of compound **9** in the KPC-2 active site. The hydrophobic and H-bond acceptor Molecular Interaction Fields are represented as yellow and red contours, respectively.

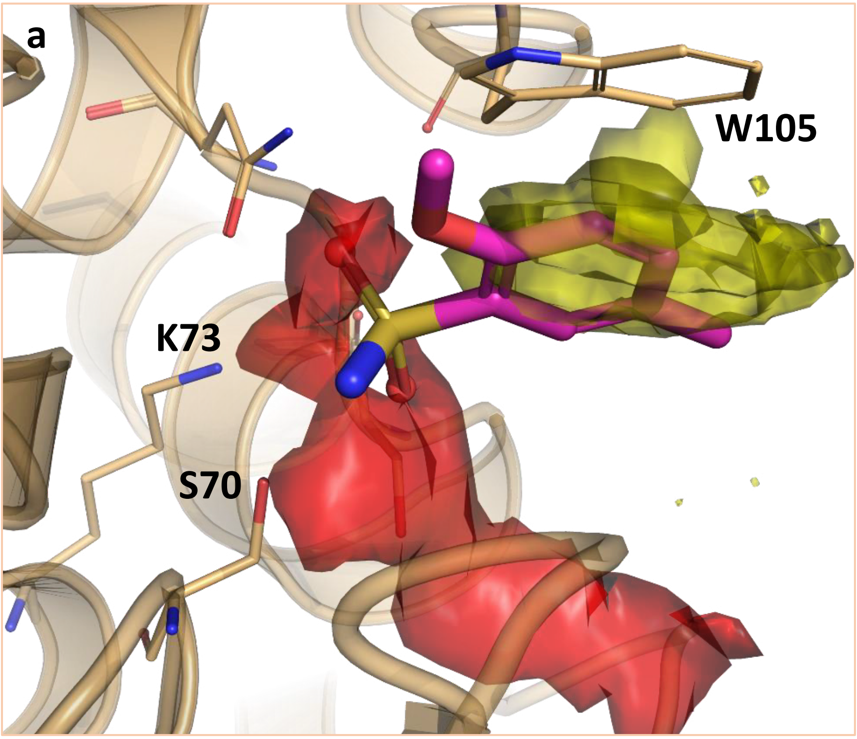

Figure S7. Docking pose of compound **7** in the CTX-M-15 active site. The hydrophobic and H-bond acceptor Molecular Interaction Fields are represented as yellow and red contours, respectively.

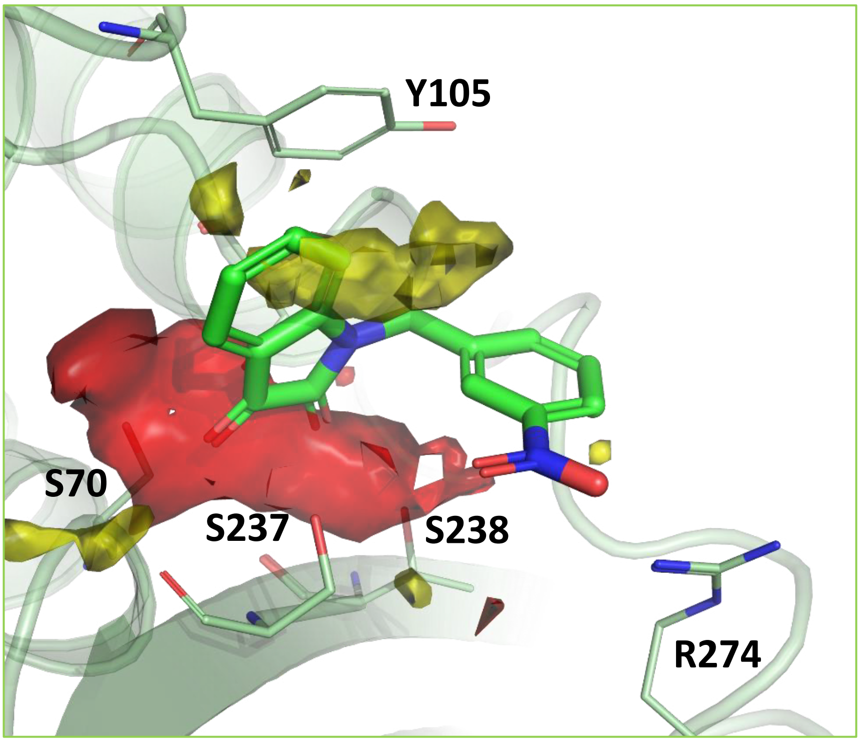

Figure S8. Docking pose of compound **54** in the AmpC active site. The hydrophobic and H-bond acceptor Molecular Interaction Fields are represented as yellow and red contours, respectively.

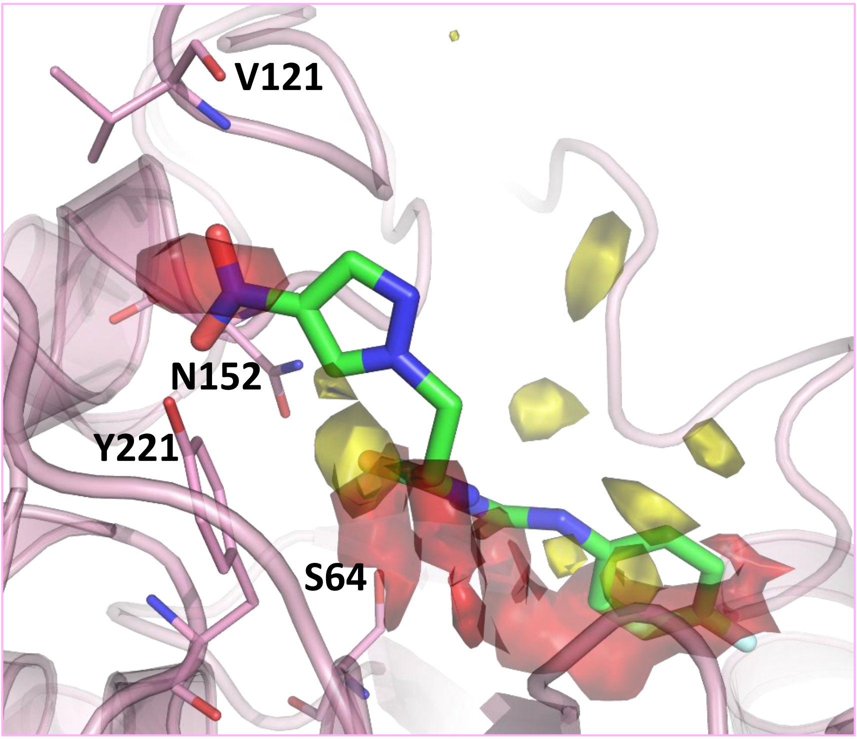

Figure S9. Superimposition of NDM-1 (cyan) and VIM-2 (grey) active sites (PDB codes 3q6x and 2yz3, respectively). **a.** Loops L3 and L10, located at the entrance of the binding site, are labelled. **b.** NDM-1 and VIM-2 active site close-up. Residues are numbered according to the BBL consensus numbering scheme.

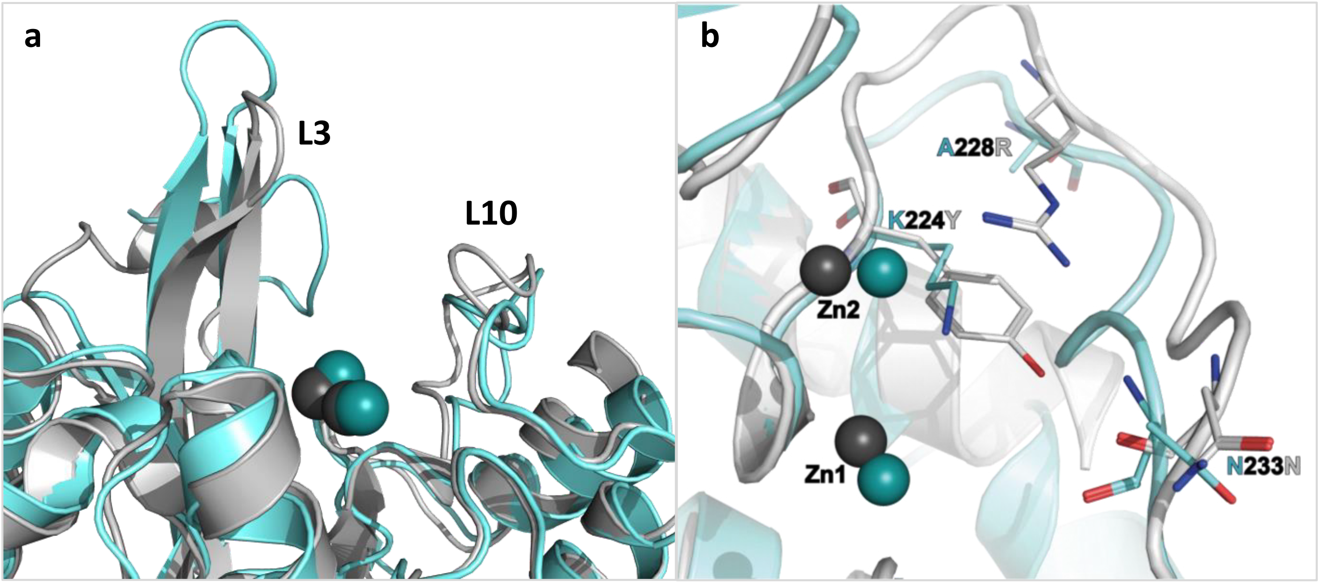

Figure S10. Superimposition of the KPC-2 (orange) and CTX-M-15 (green) binding sites (PDB code 3dw0 and 4hub, respectively). The proteins are represented as cartoons. The details of the binding sites are shown in the inset. The residues are displayed as capped sticks and those that differ in the two proteins are labelled.

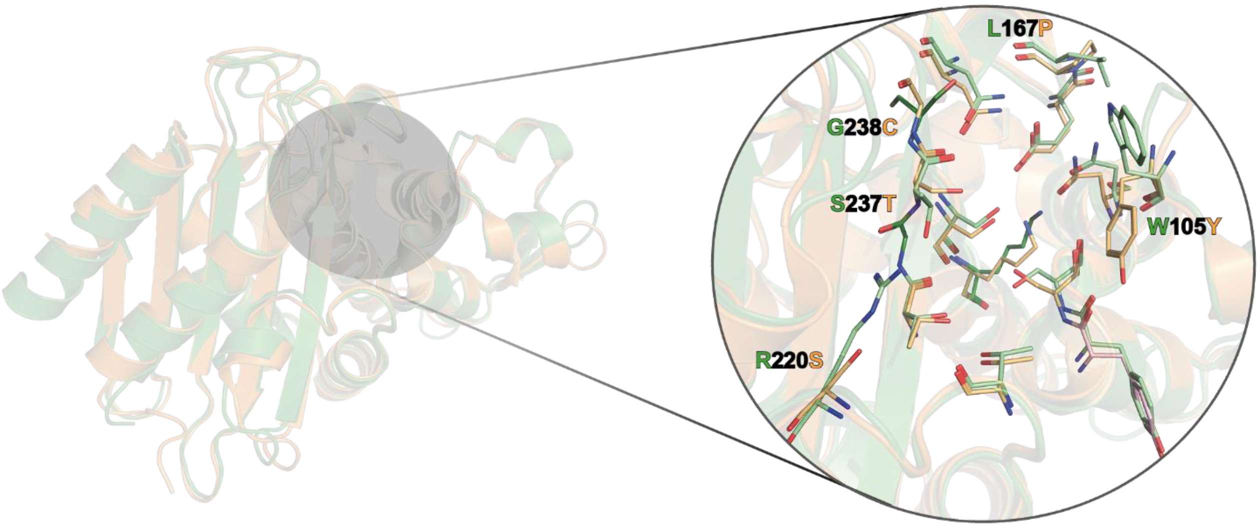

Figure S11. Docking poses of compound **59** in NDM-1 (**a**), VIM-2 (**b**), KPC-2 (**c**) and CTX-M-15 (**d**). The hydrophobic and H-bond acceptor Molecular Interaction Fields are represented as yellow and red contours, respectively. Residues are numbered according to the BBL consensus numbering scheme.

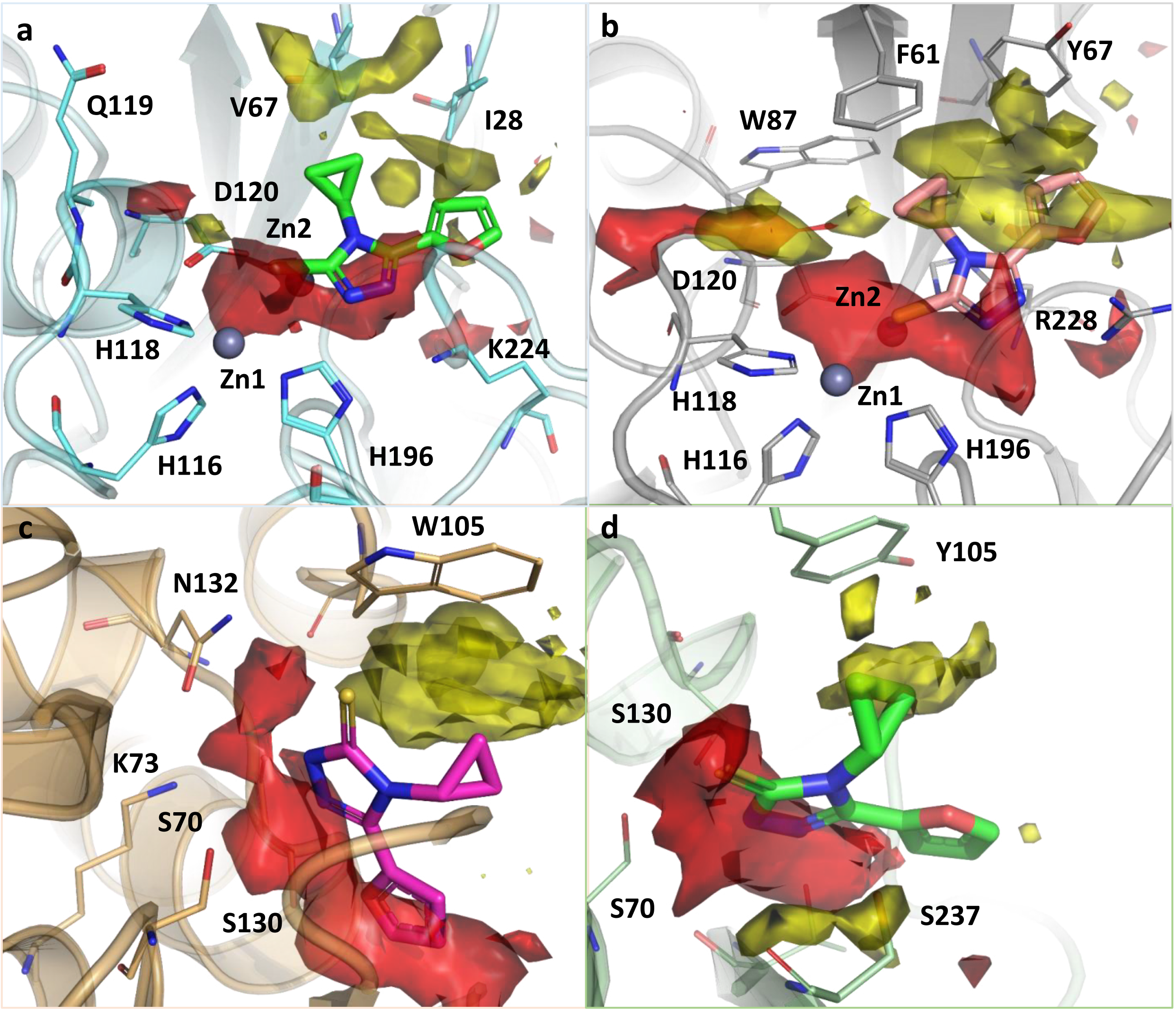

Figure S12. Superposition of MBLs NDM-1 and VIM-2 (**a**) and of SBLs KPC-2, CTX-M-15, KPC-2, AmpC (**b**), binding to compound **59**. The proteins are coloured according to the previous pictures: NDM-1 blue, VIM-2, grey, KPC-2 orange, CTX-M-15 green, AmpC pink. The proteins are shown as cartoons, the compound as capped sticks, the residues lining the pockets are labelled according to the protein colour. Residues are numbered according to the BBL consensus numbering scheme.

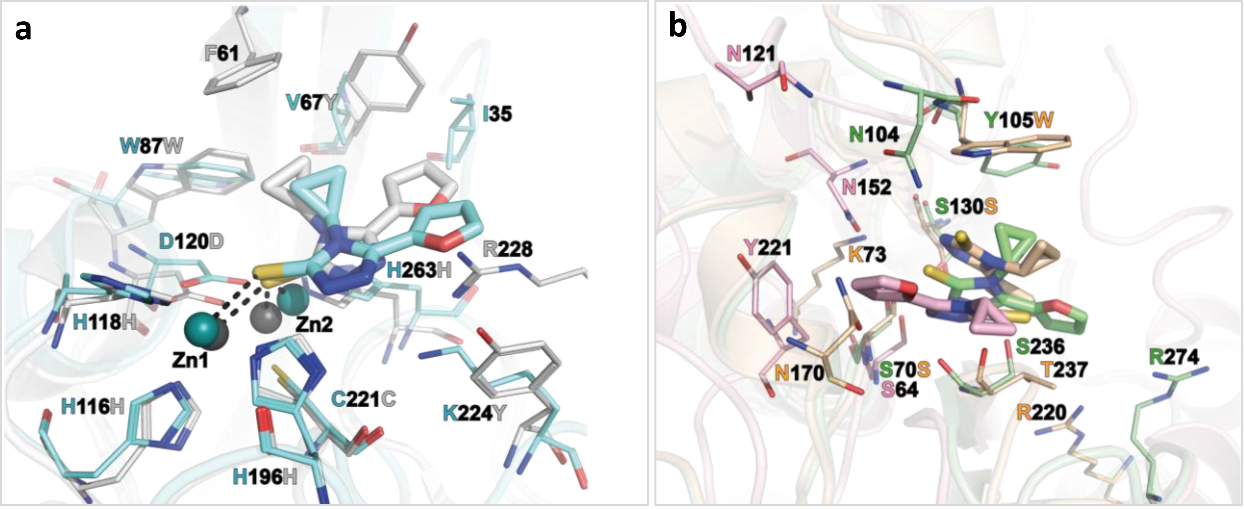

Table S4. Data collection and refinement statistics for the VIM-2-compound 24 binary complex.

	VIM2-compound 24
**Wavelength**	1.001
**Resolution range**	39.53 - 1.6 (1.657 - 1.6)
**Space group**	C 1 2 1
**Unit cell**	103.618 79.0602 68.2075 90 131.167 90
**Total reflections**	206054 (9979)
**Unique reflections**	54492 (5436)
**Multiplicity**	3.8 (3.7)
**Completeness (%)**	99.79 (99.93)
**Mean I/sigma(I)**	8.2 (1.9)
**Wilson B-factor**	17.30
**R-merge**	0.084 (0.553)
**R-meas**	0.099 (0.646)
**R-pim**	0.049 (0.330)
**CC1/2**	0.995 (0.739)
**Reflections used in refinement**	54490 (5436)
**Reflections used for R-free**	2726 (291)
**R-work**	0.1766 (0.2441)
**R-free**	0.2197 (0.2602)
**Number of non-hydrogen atoms**	4128
**macromolecules**	3559
**ligands**	62
**solvent**	507
**Protein residues**	464
**RMS(bonds)**	0.011
**RMS(angles)**	1.77
**Ramachandran favored (%)**	97.82
**Ramachandran allowed (%)**	1.74
**Ramachandran outliers (%)**	0.44
**Rotamer outliers (%)**	2.13
**Clashscore**	4.38
**Average B-factor**	21.47
**macromolecules**	19.98
**ligands**	25.69
**solvent**	31.40

Statistics for the highest-resolution shell are shown in parentheses.

Table S5. Data collection and refinement statistics for the NDM-1-compound 31 binary complex.

	NDM-1-compound 31
Wavelength	1.001
Resolution range	42.38 - 1.332 (1.38 - 1.332)
Space group	P 21 21 21
Unit cell	69.678 73.665 77.501 90 90 90
Total reflections	171702 (63904)
Unique reflections	89075 (8196)
Multiplicity	1.9 (2.0)
Completeness (%)	97.4 (93.8)
Mean I/sigma(I)	10.3 (2.1)
R-merge	0.040 (0.251)
R-meas	0.057 (0.355)
R-pim	0.040 (0.251)
CC1/2	0.999 (0.714)
Reflections used in refinement	89070 (8540)
Reflections used for R-free	4611 (493)
R-work	0.1810 (0.5067)
R-free	0.2032 (0.5238)
CC(work)	0.961 (0.602)
CC(free)	0.951 (0.545)
Number of non-hydrogen atoms	3909
macromolecules	3519
ligands	44
solvent	346
Protein residues	460
RMS(bonds)	0.011
RMS(angles)	1.81
Ramachandran favored (%)	98.46
Ramachandran allowed (%)	1.54
Ramachandran outliers (%)	0.00
Rotamer outliers (%)	3.63
Clashscore	5.41
Average B-factor	19.04
macromolecules	18.15
ligands	31.09
solvent	26.60

Statistics for the highest-resolution shell are shown in parentheses.

Scheme S1. ΔVIM-2 synthetic gene sequence

```
                                  V   D   S   S   G   E   Y   P   T   V   S   E   I
  1 CGC GAA CAG ATT GGA GGT TCG GTT GAC TCT TCC GGC GAA TAT CCT ACC GTG TCT GAG ATT
      P   V   G   E   V   R   L   Y   Q   I   A   D   G   V   W   S   H   I   A   T
 61 CCG GTT GGC GAA GTA CGG TTA TAC CAG ATT GCA GAT GGG GTG TGG AGT CAT ATT GCC ACC
      Q   S   F   D   G   A   V   Y   P   S   N   G   L   I   V   R   D   G   D   E
121 CAA TCC TTC GAT GGC GCC GTG TAT CCG AGC AAT GGC CTG ATT GTT CGT GAT GGC GAT GAA
      L   L   L   I   D   T   A   W   G   A   K   N   T   A   A   L   L   A   E   I
181 CTG CTG CTG ATC GAT ACC GCG TGG GGT GCG AAG AAC ACC GCA GCC CTT CTG GCA GAA ATC
      E   K   Q   I   G   L   P   V   T   R   A   V   S   T   H   F   H   D   D   R
241 GAG AAA CAG ATC GGT CTG CCG GTG ACT CGT GCG GTA AGC ACG CAC TTC CAC GAT GAC CGT
      V   G   G   V   D   V   L   R   A   A   G   V   A   T   Y   A   S   P   S   T
301 GTT GGT GGC GTG GAC GTG CTT CGC GCT GCT GGT GTT GCG ACC TAT GCG AGT CCC AGC ACA
      R   R   L   A   E   V   E   G   N   E   I   P   T   H   S   L   E   G   L   S
361 CGT CGC TTA GCC GAA GTG GAA GGT AAC GAG ATT CCG ACC CAT TCA CTG GAA GGG TTA AGC
      S   S   G   D   A   V   R   F   G   P   V   E   L   F   Y   P   G   A   A   H
421 AGC TCA GGC GAT GCC GTT CGC TTT GGC CCA GTG GAG CTG TTT TAC CCA GGT GCT GCA CAC
      S   T   D   N   L   V   V   Y   V   P   S   A   S   V   L   Y   G   G   C   A
481 TCC ACT GAC AAC TTG GTC GTC TAC GTA CCG AGT GCA AGC GTC CTG TAT GGC GGA TGC GCC
      I   Y   E   L   S   R   T   S   A   G   N   V   A   D   A   D   L   A   E   W
541 ATC TAC GAA CTG TCA CGC ACA TCT GCG GGG AAT GTC GCG GAT GCG GAT TTG GCT GAA TGG
      P   T   S   I   E   R   I   Q   Q   H   Y   P   E   A   Q   F   V   I   P   G
601 CCG ACG TCG ATC GAG CGC ATT CAA CAG CAT TAT CCC GAA GCG CAG TTT GTG ATT CCG GGA
      H   G   L   P   G   G   L   D   L   L   K   H   T   T   N   V   V   K   A   H
661 CAT GGA CTG CCT GGT GGT TTG GAC CTC CTC AAA CAC ACG ACG AAC GTC GTA AAA GCC CAT
      T   N   R   S   V   V   E   *
721 ACC AAT CGC TCG GTT GTG GAA TGA GGT ACC TAA TAG AGC GGC CGC CAC
```
